# A biphasic epigenetic switch controls immunoevasion, virulence and niche adaptation in non-typeable *Haemophilus influenzae*

**DOI:** 10.1038/ncomms8828

**Published:** 2015-07-28

**Authors:** John M. Atack, Yogitha N. Srikhanta, Kate L. Fox, Joseph A. Jurcisek, Kenneth L. Brockman, Tyson A. Clark, Matthew Boitano, Peter M. Power, Freda E.-C. Jen, Alastair G. McEwan, Sean M. Grimmond, Arnold L. Smith, Stephen J. Barenkamp, Jonas Korlach, Lauren O. Bakaletz, Michael P. Jennings

**Affiliations:** 1 Institute for Glycomics, Griffith University, Gold Coast, Queensland 4222, Australia; 2 School of Chemistry and Molecular Biosciences, The University of Queensland, St Lucia, Brisbane, Queensland 4072, Australia; 3 Center for Microbial Pathogenesis, The Research Institute at Nationwide Children’s Hospital and The Ohio State University College of Medicine, Columbus, Ohio 43205, USA; 4 Pacific Biosciences, 1380 Willow Road, Menlo Park, California 94025, USA; 5 Institute of Molecular Bioscience, The University of Queensland, St Lucia, Brisbane, Queensland 4072, Australia; 6 Center for Global Infectious Disease Research, Seattle Children's Research Institute, Seattle, Washington 98105, USA; 7 Department of Pediatrics, Saint Louis University School of Medicine, and the Pediatric Research Institute, Cardinal Glennon Children’s Medical Center, Saint Louis, Missouri 63104, USA

## Abstract

Non-typeable *Haemophilus influenzae* contains an N^6^-adenine DNA-methyltransferase (ModA) that is subject to phase-variable expression (random ON/OFF switching). Five *modA* alleles, *modA2*, *modA4, modA5*, *modA9* and *modA10*, account for over two-thirds of clinical otitis media isolates surveyed. Here, we use single molecule, real-time (SMRT) methylome analysis to identify the DNA-recognition motifs for all five of these *modA* alleles. Phase variation of these alleles regulates multiple proteins including vaccine candidates, and key virulence phenotypes such as antibiotic resistance (*modA2*, *modA5*, *modA10*), biofilm formation (*modA2*) and immunoevasion (*modA4*). Analyses of a *modA2* strain in the chinchilla model of otitis media show a clear selection for ON switching of *modA2* in the middle ear. Our results indicate that a biphasic epigenetic switch can control bacterial virulence, immunoevasion and niche adaptation in an animal model system.

## Abstract (web summary)

Non-typeable *Haemophilus influenzae*, which causes ear and lung infections, has a DNA methyltransferase encoded by alternative alleles that are subject to random ON/OFF switching. Here, Atack *et al.* show that this epigenetic switch controls the expression of key proteins involved in virulence.

## Graphical abstract

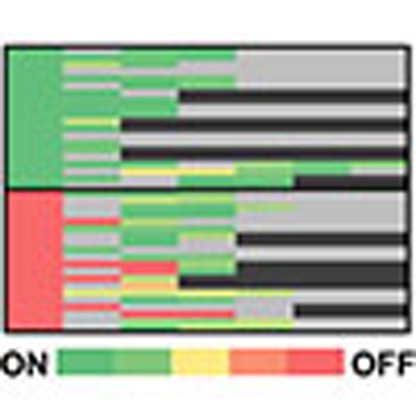

Non-typeable *Haemophilus influenzae* (NTHi) is a significant bacterial pathogen commonly associated with paediatric infection as a predominant cause of otitis media (OM) or middle ear infection. While NTHi contributes to ∼40% of all cases of acute OM, it remains the major aetiological agent of chronic OM, recurrent acute OM and OM treatment failure^1^. It is also a major cause of community-acquired pneumonia and exacerbations of chronic obstructive pulmonary disease. With the rising prevalence of antimicrobial resistance, the success rate in the treatment of NTHi has recently declined. There is no vaccine to prevent infection by NTHi.

Phase variation is the high frequency reversible ON/OFF switching of gene expression, and is a common feature of many virulence determinants expressed by bacterial pathogens^2^,^3^,^4^,^5^. While phase variation is typically associated with genes that encode surface structures, several host-adapted bacterial pathogens, including NTHi, have DNA methyltransferases (*mod* genes) associated with type III restriction modification systems that are subject to phase-variable expression. Simple tandem DNA repeats have been shown to mediate phase variation of *mod* genes by rapid reversible loss or gain of a repeat unit leading to frame shifts and ON/OFF switching of expression^6^,^7^,^8^,^9^,^10^.

The variable central region of Mod methyltransferases contains the DNA-recognition domain (DRD; see Fig. 1e) that dictates methylation-sequence specificity^11^,^12^,^13^. In *H. influenzae* strain Rd, our previous work has demonstrated that random switching of the *modA1* gene controls expression of multiple genes via differential methylation of the genome in the *modA1* ON and OFF states^10^. This novel genetic system, termed the phasevarion (phase-variable regulon) regulates gene expression in four other important human pathogens: *Neisseria gonorrhoeae, Neisseria meningitidis*^7^, *Helicobacter pylori*^9^ and *Moraxella catarrhalis*^14^. Differences in the DRD have previously been observed in a genetically diverse collection of *H. influenzae* strains and these differences define 20 distinct *mod* alleles (*modA1 to modA20*)^6^,^15^. Different DRDs are proposed to recognize and methylate different target sequences. Our studies with pathogenic *Neisseria,* which also contains *modA*, confirmed that different *modA* alleles methylate different target sequences and thereby regulate different sets of genes, that is, have distinct phasevarions. Conversely, strains that harbour the same *modA* allele regulate the same phasevarion of genes^7^,^16^.

In this study, we sought to assess the role that candidate NTHi phasevarions may play in gene expression, immunoevasion and in the pathogenesis of NTHi during experimental OM. Five *modA* alleles are present in over two-thirds of all isolates of NTHI. We demonstrate that phase variation of all five of these *modA* alleles controls gene expression differences in all the strains studied. These expression differences include outer-membrane proteins and key vaccine candidates. Biphasic switching of specific *modA* alleles also influences fitness in opsonophagocytic killing assays and in susceptibility to antibiotics. In an *in vivo* animal model, we demonstrate, for the first time, direct selection for a particular state of *modA* expression in the major phase variable *modA* allele present in NTHi, *modA2*.

## Results

### Distribution of *modA* alleles in NTHi isolates

**Figure 1:**
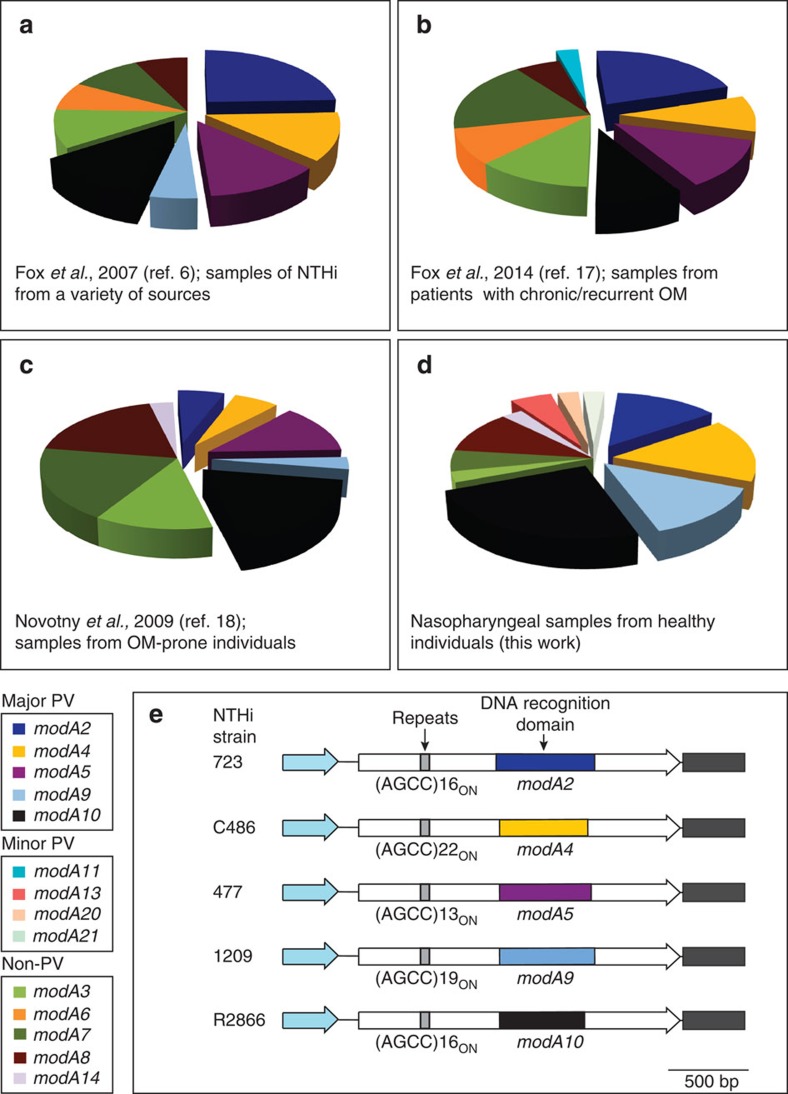
Analysis of NTHi strain collections to ascertain the proportion of isolates containing phase variable *modA* alleles. (**a**–**d**) Presents the four separate strain collections that were analysed to ascertain the distribution of phase variable *modA* alleles in NTHi populations; (**a**) collection containing a broad selection of NTHi isolates from a variety of sources (for example, culture collections, OM-prone individuals)^6^; (**b**) samples of paired isolates of NTHi isolated from patients presenting with chronic/recurrent OM^17^; (**c**) sample collection of NTHi isolates taken from OM-prone individuals^18^; (**d**) NTHi isolates from the nasopharynx of healthy children (collected as part of this study); and (**e**) the five *modA* alleles most commonly associated with OM—*modA2, modA4, modA5, modA9* and *modA10.* Each *modA* gene is represented as a white arrow, with the DRD represented by a coloured box that matches the colour in panels **a**–**d**. The black box to the right of each gene represents the 5′ region of the downstream gene (an inactive restriction endonuclease). The blue arrow to the left of each *modA* gene represents the 3′ end of the gene upstream of each *modA* (in all the cases, a ribonuclease, *rnhB*). Details of the strain collections used in **c** and **d** are presented in Supplementary Table 1.

We conducted a detailed analysis examining the *modA* allele frequency in a diverse set of NTHi isolates taken from healthy individuals and OM patients (Fig. 1). The *modA* alleles *modA3, modA6*, *modA7*, *modA8* and *modA14* do not contain tetranucleotide repeats, and thereby do not phase vary in expression. In a previous survey^6^, we reported that ∼70% of NTHi strains contain a phase variable *modA* allele (*n*=41; Fig. 1a). Of all the *modA* alleles, *modA2* was the most prevalent, being found in almost a quarter of all isolates (24%).

We then analysed *modA* allele distribution in three further strain collections (Fig. 1). The first collection contained paired isolates, isolated from the nasopharynx and the middle ear, from children presenting with chronic and/or recurrent OM (Fig. 1b)^17^. This collection contained 27 pairs of isolates: 16 pairs were collected from 1982 to 1986 and 11 pairs were collected from 2004 to 2006. Almost two-thirds of strains (59.3%) within this particular collection contain a phase variable *modA* allele (*n*=33), with *modA2* most prevalent (22% of all isolates). The second strain collection contained clinical isolates from OM-prone children (Fig. 1c)^18^. Of these 34 separate isolates (25 recovered from nasopharyngeal swabs and 9 recovered from middle ear effusions), 65% contained a phase variable *modA* allele. The most common was *modA10* (19%). As NTHi is frequently isolated from the upper respiratory tract, we collected samples and analysed *modA* allele distribution in isolates from the nasopharynges of healthy children (Fig. 1d), with a reported colonization rate of ∼80% (ref. 19). In addition, a correlation between NTHi colonization frequency and the incidence of recurrent OM in children has been suggested^20^. We observed that the predominant *modA* alleles in healthy nasopharyngeal isolates were *modA10* (24%), *modA4* (16%), *modA2* (14%) and *modA9* (14%) (Fig. 1d). These four *modA* alleles are all phase variable, and alone comprise over two-thirds of all alleles from this collection. One new *modA* allele was also identified in this study in strain 515 (Supplementary Table 1). We have designated this new allele as *modA21*.

Taken together, this analysis of four distinct strain collections shows that not only are phase variable *modA* alleles frequently associated with disease, they are also prevalent in NTHi isolated from healthy individuals. From all the four studies, phase variable *modA* alleles are present in approximately two-thirds of all the isolates (*n*=135), with the *modA2* and *modA10* alleles, the two most common overall, at 17.2 and 15.4%, respectively. Based on these surveys, we selected the five *modA* alleles most commonly associated with OM—*modA2, 4, 5, 9* and *10*—for further analysis in this study (Fig. 1e).

### Generation of natural *modA* ON and OFF strains

**Figure 2:**
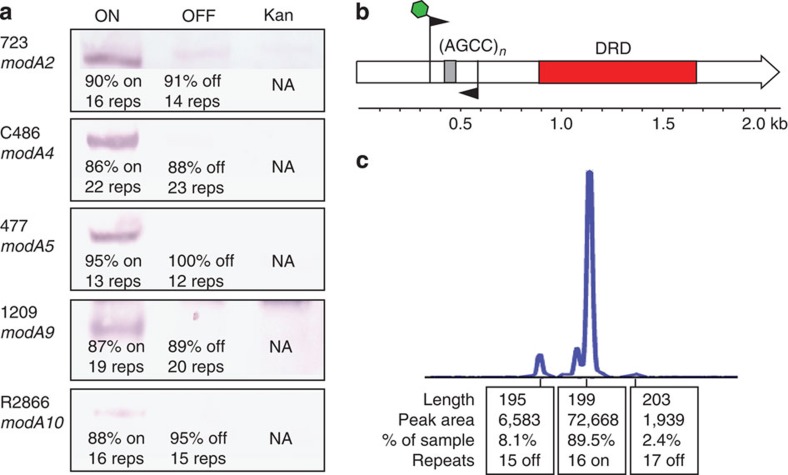
Western blots showing ModA presence in *modA* ON OFF and kanamycin knockout strains and an example of the fragment analysis methodology. (**a**) Western blot analysis using ON/OFF/*kan* cells from all the five *modA* alleles; anti-*modA* primary antibody at 1:5,000; AP conjugated anti-rabbit secondary at 1:20,000. A *modA* band is only present in those cells possessing the ON variant of each strain. The number of repeats (reps) represents the major number of repeats present in that particular isolate, and the percentage ON of that particular strain is noted underneath each relevant blot. Full western blots are presented in Supplementary Fig. 4; (**b**) an illustration of the methodology used during fragment analysis to ascertain *modA* ON/OFF state and percentages of cell populations. A 6-carboxyflourescein (FAM)-labelled forward primer (green hexagon) and unlabelled reverse primer was used to generate a PCR product over the repeat tract (AGCC_*n*_; grey box) that was analysed using GeneScan technology; and (**c**) an example trace produced by GeneScan analysis on a representative genomic sample from an NTHi strain containing a phase variable *modA* gene.

We generated *modA* natural ON- and OFF-enriched populations (>90% ON or OFF) from the five NTHi strains containing the modA2, 4, 5, 9 and 10 alleles (Fig. 1e). All ON strains contain a number of AGCC repeats that places the *modA* open reading frame (ORF) in frame, and therefore express a full-length functional protein, as confirmed by western blot using an anti-ModA antibody (Fig. 2a). All the natural ON and OFF strains were continually verified using our well-established FAM-labelled primer PCR screen coupled to fragment length analysis (an example of the methodology and results is given in Fig. 2b,c)^6^,^10^. Natural *modA*OFF strains contain a number of AGCC repeats that place the *modA* ORF out of frame, leading to a frame shift mutation and premature termination at stop codons in the alternate reading frames (Fig. 2a). Kanamycin knockout *modA::kan* mutants were also generated in all the five NTHi strains bearing these alleles (Fig. 1e) by disruption of the *modA* gene via insertion of a kanamycin resistance cassette, as described previously^6^,^10^.

### Methylome analysis

Single molecule, real-time (SMRT) sequencing^21^ was carried out to determine the complete genomes and identify the methylation recognition sequence of these five distinct ModA N^6^-adenine methyltransferases. DNA isolated from each *modA*ON and *modA*::*kan* pair was sequenced and analysed. By comparing the methylomes of the *modA*ON with the *modA::kan* knockout mutants, we were able to identify the motif methylated by the ModA methyltransferases of ModA4, 5, 9, 10 (Table 1). The ModA2 motif was identified by heterologous expression of ModA2 in *Escherichia coli*, as described previously^22^. ModA2, ModA5, ModA9 and ModA10 all have prototypical type III methyltransferase recognition sequences (Table 1), in that they methylate an adenine residue on the *N*^6^ position in a 5 base pair (5 bp) non-palindromic sequence^23^,^24^. The recognition sequence of ModA4 was different, instead recognizing a four base sequence: 5'-CG(^m6^A)G-3′ (Table 1). In accordance with REBASE^25^ naming conventions, we have given the ModA2, ModA4, ModA5, ModA9 and ModA10 methyltransferases the designations M.Hin723I, M.HinC486I, M.Hin477I, M.Hin1209I and M.Hin2866I, respectively. Summary data from the SMRT methylome analysis of the five *modA* ON/OFF pairs is shown in Supplementary Fig. 1. Complete closed genome sequences for each of the five strains containing these *modA* alleles were also generated (NTHi strains 723, C486, 477, 1209 and R2866; the R2866 genome sequence had already been deposited in NCBI Genbank; accession number CP002277). Accession codes for the complete annotated genomes of NTHi strains 723, C486, 477 and 1209 are listed in the ‘Accession codes’ section.

### Distribution of ModA sites in NTHi genomes

Bioinformatic analysis of the recognition sequences of ModA2, 4, 5, 9, 10 showed that these sites are widely and evenly distributed in their respective genomes (see Supplementary Fig. 1). ModA4 occurs much more frequently than ModA2, 5, 9 or 10, as is expected for a 4 bp recognition sequence compared with 5 bp recognition sequences. For ModA2, A4, A5 and A9, sites appear to be equally distributed between coding and non-coding regions of the genome. For ModA10, 69.1% of sites are present in non-coding regions, compared with only 30.9% of ModA10 sites in coding regions. Further, there are far fewer ModA10 sites in the R2866 genome than would be expected by chance (1,244 observed/2,714 expected=45.8%; Supplementary Fig. 1). Other ModA recognition sequences occurred at frequencies close to that predicted by chance: ModA2 (91.2%), ModA4 (79%) ModA5 (104.7%), ModA9 (94%).

### *modA* ON/OFF pairs show outer-membrane protein expression differences

**Figure 3:**
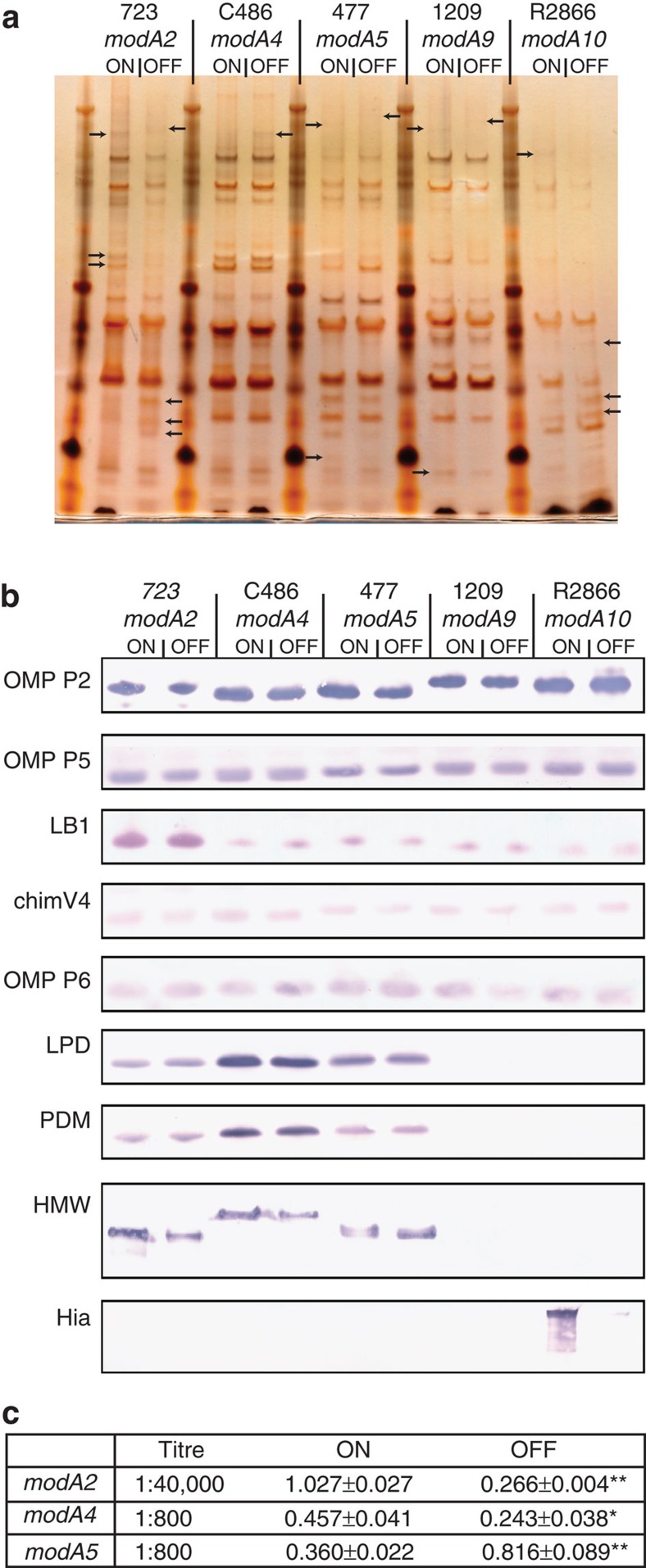
Analysis of outer-membrane protein (OMP) preparations. (**a**) Silver-stained gel of each *modA* ON/OFF pair; (**b**) western blots using antibodies against currently investigated vaccine candidates. Full western blots are presented in Supplementary Fig. 5; (**c**) ELISA results for HMW in *modA2*, *modA4* and *modA5* ON/OFF strain pairs using primary antibody AD6. Values presented are mean values±s.d. Each experiment was carried out in triplicate. *P* values were calculated using Student’s *t*-test based on specific titres in the linear range of the response curve (*=<0.01; **=<0.001). Full ELISA data are presented in Supplementary Data 1.

To determine whether *modA* switching altered the expression of outer-membrane proteins (OMPs), outer-membrane samples were separated using Bis-Tris PAGE gels and silver stained (Fig. 3a) to explore whether any gross protein differences existed within each *modA* ON/OFF strain pair. Clear differences could be visualized within the OMP profile of each *modA* ON/OFF pair (noted by arrows in Fig. 3a), indicating that *modA* phase variation influences the OMP profile, and may therefore influence immunoevasion, pathogenesis and virulence.

### Vaccine candidate expression in *modA* ON/OFF strain pairs

OMP preparations from *modA* ON and OFF strains were studied using western blot with primary antibodies raised against several well-studied vaccine candidates (Fig. 3b): major outer membrane proteins P2 (ref. 26), P5 (refs 27, 28) and P6 (refs 29, 30); lipoprotein D^31^ and its deacylated derivative PDM^31^,^32^; the high molecular weight (HMW) proteins^33^,^34^; and the adhesin Hia^35^. Three different antisera were used to probe for relative expression of OMP P5 (anti-P5, anti-LB1 and anti-chimV4), as these sera contained antibodies specific to unique epitopes of the protein^27^,^28^,^36^ (Fig. 3b).

We saw an expression difference of OMP P6 in the *modA9* ON/OFF pair, with a greater level of OMP P6 seen in *modA9*ON relative to *modA9*OFF cells (Fig. 3b). Lipoprotein D showed stable expression independent of *modA* status in NTHi strains 723 (*modA2*), C486 (*modA4*) and 477 (*modA5*) (Fig. 3b). We could not detect the presence of lipoprotein D in NTHi strains 1209 or R2866, regardless of *modA* status (Fig. 3b). The analysis of our NTHi strain 1209 SMRT-generated genome revealed that the gene-encoding lipoprotein D (*glpQ*) is truncated in this strain (1209_00054) to just the carboxy (C)-terminal portion (∼65 residues). In strain R2866, a full-length gene is present (R2866_1787), but it appears to not be expressed at a level detectable by western blotting.

Expression of HMW1/2A was greater in OMPs prepared from *modA2*ON versus *modA2*OFF; *modA4*ON versus *modA4*OFF; and *modA5*OFF versus *modA5*ON (Fig. 3b). In these latter two examples, a single protein band was clearly more prevalent in *modA4*ON and *modA5*OFF, when compared with their respective partners (Fig. 3b). However, in *modA2*ON, a higher MW band was more abundant in *modA2*ON compared with *modA2*OFF, and a lower MW band was visible only in *modA2*ON, implying that both HMW1A and HMW2A are present in greater amounts in OMP preparations from NTHi *modA2*ON when compared with *modA2*OFF (Fig. 3b). Apparent differences in HMW expression in western blot results were verified using whole-cell enzyme-linked immunosorbent assays (ELISAs) to quantitate the relative expression of HMW1/2A. Statistical significance was calculated using Student’s *t*-test, and significant differences were observed for these three ON/OFF strain pairs (Fig. 3c).

As the genes encoding HMW1A and HMW2A proteins contain variable heptanucleotide repeats in their promoter region that are also reported to influence gene expression^4^,^37^, we sequenced across this repeat tract in all the three strain pairs where a difference in HMW expression was evident. This analysis showed that the differences in repeat tract length in each ON/OFF pair were not responsible for the expression changes observed (Supplementary Fig. 2). The literature states that the longer the repeat tract, the lesser the expression level of HMW^37^. As the repeat tracts of HMW1 and HMW2 are of identical or similar length within each strain pair where differences are seen, it is likely that expression differences are ModA-dependent, and not due to HMW phase variation.

Finally, our analysis of the adhesin Hia revealed that the *hia* gene was only present in the genome of NTHi strain R2866 (R2866_0725) containing the *modA10* allele (Fig. 3b). Hia was expressed at a greater level in *modA10*ON relative to *modA10*OFF (Fig. 3b). However, our subsequent analysis showed that this difference was owing to *modA10*-independent phase variation event in a poly-T tract in the Hia promoter region^38^.

### iTRAQ proteomics analysis of *modA* ON/OFF strain pairs

To further define the impact of *modA* ON/OFF status on the expression of existing and potential vaccine candidates, as well as the OMP profile as a whole, preparations of OMPs used in silver staining and western blotting (Fig. 3) were characterized using iTRAQ 1D nanoLC ESI MS/MS. All the data have been deposited to the ProteomeXchange Consortium^39^ via the PRIDE partner repository, with the data set identifier PXD002210. Proteins with either >1.5- or <0.65-fold expression differences between ON/OFF pairs when comparing two biological replicates of each OMP preparation are reported (Table 2). Expression changes in OMPs were seen in the *modA2*, *modA4, modA5* and *modA10* ON/OFF strain pairs.

Several OMPs involved in the sequestration of iron and haem were downregulated in *modA2*ON (Table 2). This included HxuC and HxuB (723_01435 and 723_01434; a haem/haemopexin utilization and haem/haemopexin-binding OMP, respectively), transferrin-binding protein 1 (723_00620), a hemin receptor (HemR, also annotated as HxuC2; 723_01596); and a major ferric iron-binding protein (HitA, 723_01615). This analysis also revealed OMP P6 to be differentially regulated in the *modA2* pair (Table 2), a finding not evident from our western blotting analysis (Fig. 3b), but having implications for its use as a suitable vaccine antigen.

HMW2B is an outer membrane-associated protein responsible for the translocation of the adhesins HMW1/2A to the cell surface^40^. The increase in an accessory protein in *modA2*ON with specificity for both HMW1/2A proteins would explain the increase in both HMW-A proteins in *modA2*ON seen in our western blotting with the *modA2* strain pair (Fig. 3b). NanM, a sialic acid mutarose, was also present in greater amounts in the outer membrane of *modA2*ON cells (Table 2).

### Phase variation of *modA* influences susceptibility to antibiotics

Phasevarion-mediated differential susceptibility to antibiotics has been reported in other human-adapted pathogens^41^, so we sought to investigate whether this was also the case for our five *modA* ON/OFF pairs in NTHi. Minimum inhibitory concentration (MIC) analysis showed that phase-variable expression of *modA2*, *modA5* and *modA10* led to 2-fold changes in susceptibility to ampicillin, erythromycin and gentamicin, respectively (Supplementary Table 2). These findings suggest that gene regulation through DNA methylation is an additional element that may contribute to antibiotic susceptibility.

### ModA4 mediates evasion of opsonophagocytic killing

**Figure 4:**
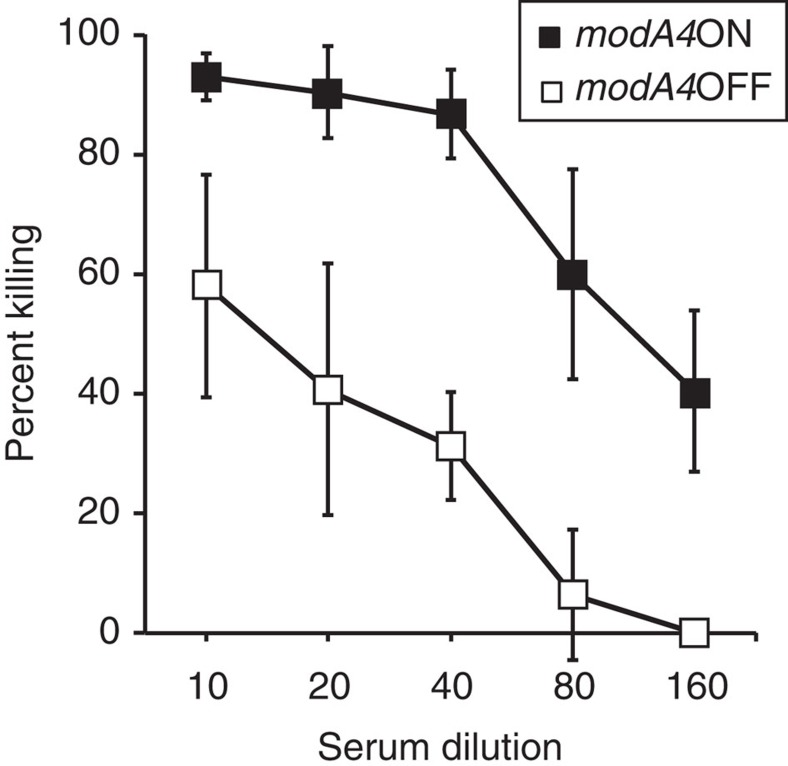
Opsonophagocytic killing curves using strain C486 *modA4* ON/OFF strain pair. Assays were carried out using guinea pig antiserum raised against purified HMW proteins from NTHi strain 12, with NTHi strain C486 harbouring the *modA4* ON/OFF pair. Three independent assays were carried out per strain, with error bars representing one s.d. Statistical significance was calculated using Student’s *t*-test, and was <0.05 at all dilutions of antiserum. Individual *P* values—1:10=0.034; 1:20=0.0185; 1:40=0.0012; 1:80=0.009; 1:160=0.0065.

Differential expression of HMW protein had been observed in strain C486, containing the *modA4* phasevarion. Antiserum was available that would recognize the HMW protein in this strain. We tested our C486 *modA4* ON/OFF strain pair in an opsonophagocytic killing assay^42^. The *modA4*ON strain was significantly (*P*<0.05 at all the data points; calculated using Student’s *t*-test) more susceptible to killing at every dilution of antibody tested compared with *modA4*OFF (Fig. 4). No killing was observed with this strain pair using pre-immune sera from animals used to raise anti-HMW sera, nor was killing observed with antisera raised against the protein Hia (data not shown).

### Differentially expressed genes in the *modA2* phasevarion

Our analysis of samples from a wide variety of sources showed that *modA2* is the most prevalent *modA* allele present in NTHi isolates (Fig. 1), suggesting that *modA2* may have an important role in both asymptomatic colonization and disease. iTRAQ proteomic analysis also showed more differences in expression profile analysis between *modA2*ON and *modA2*OFF than the other four *modA* strain pairs. These observations could lead to a greater potential for phenotypic and virulence differences *in vivo* (Table 2). We therefore selected the *modA2* phasevarion for microarray analysis. RNA was isolated and compared from the *modA2*ON strain and the *modA2::kan* mutant. We found 36 genes with an expression ratio of 1.4-fold or greater, with 27 genes upregulated in *modA2::kan* relative to *modA2*ON and nine genes downregulated, thereby demonstrating *modA2* phase variation has a global impact on gene expression (Table 3; full results in Supplementary Table 3). Quantitative real-time PCR specific for several of the differentially expressed genes confirmed the array data (Table 3). This analysis adds further evidence to our observations using iTRAQ quantitative mass spectrometry that there is differential regulation of genes involved in iron acquisition in the *modA2* strain pair (Table 2; Table 3). For example both *hxuB* 723_01434 and *hitA* 723_1615 are identified as differentially regulated by both iTRAQ and microarray analysis. Microarray analysis revealed several additional iron-regulated genes that were not identified by iTRAQ as they are not OMPs. These include *hitB,* which encodes a cytoplasmic permease, with both HitA and HitB being essential for the utilization of iron by NTHi^43^; and *hxuA*, a secreted protein involved in the binding of haem–haemopexin. Functional HxuA and HxuB proteins are required for virulence in *H. influenzae*^44^. Also upregulated in 723 *modA2::kan* was the *yfeABCD* operon, which encodes an iron transport system^45^. This locus has homology to the *yfeABCD* locus of *Yersinia pestis,* where it is associated with virulence^46^. Several genes involved in anaerobic metabolism also showed increased expression in *modA2::kan* (Table 3). Many enzymes involved in anaerobic respiration appear to play an important role in colonization and virulence in bacterial pathogens^47^,^48^.

### Strain 723 *modA2*ON is preferentially selected *in vivo*

To determine whether the gene expression differences between the *modA2* natural ON and OFF states impacted virulence, a variant of NTHi strain 723 selected for its *modA2*ON status (∼90% ON, 22 AGCC repeats; *modA2*(22)ON) and a variant selected for its *modA2*OFF status (∼90% OFF. 24 AGCC repeats; *modA2*(24)OFF) were used as challenge strains in the well-established chinchilla model of OM^49^. These variants were free to phase-vary during the 22 days of the experiment. In two separate studies, cohorts of five chinchillas each (*n*=10 total) were challenged intranasally and transbullarly with NTHi strain 723 *modA2*(22)ON or strain 723 *modA2*(24)OFF. Middle ear fluids were collected at regular intervals to assess any differences between these strains in their ability to infect the middle ear. Samples taken directly from the left and right middle ear were immediately snap frozen in liquid nitrogen, and were not subcultured, thereby reflecting the exact ON/OFF status of the bacterial population from the site and time of sampling.

**Figure 5:**
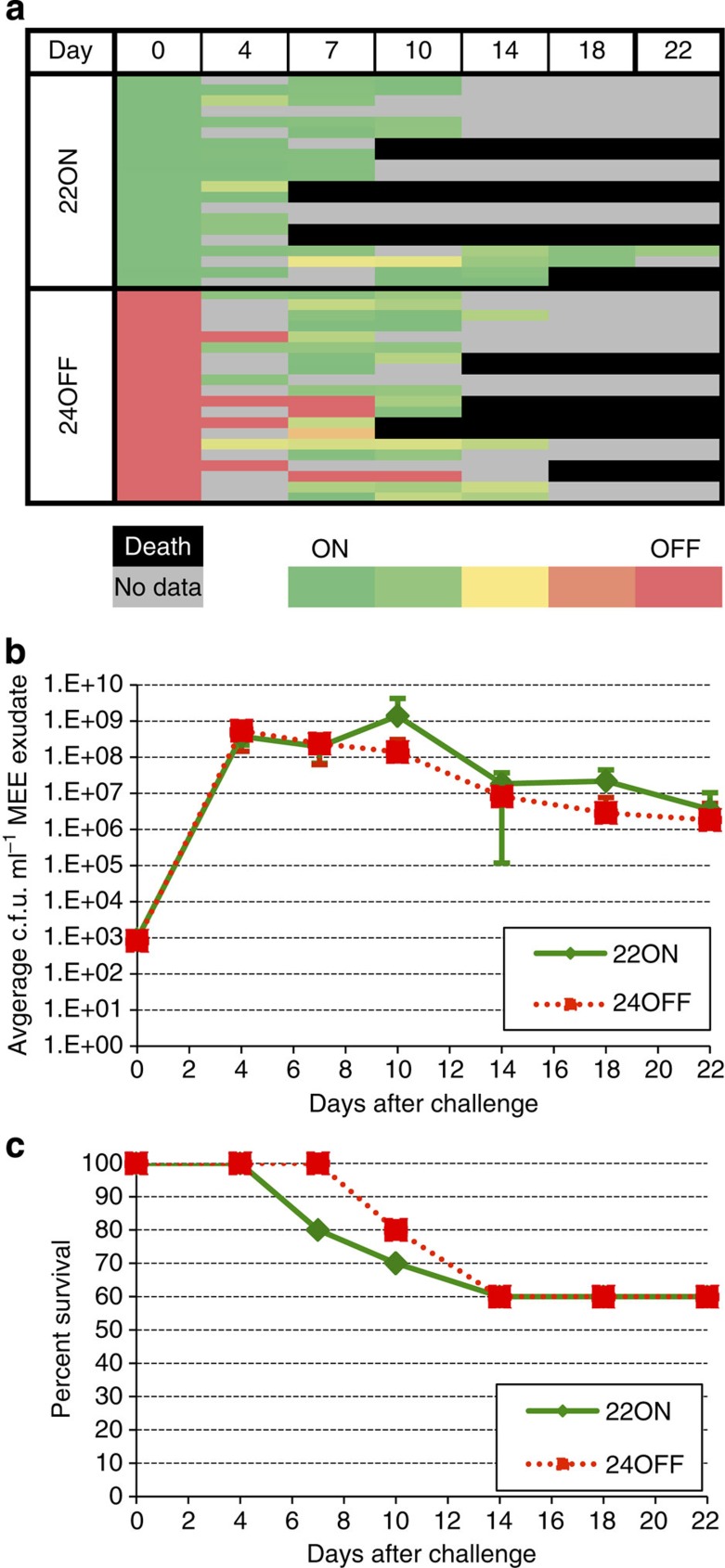
Results from chinchilla middle ear samples. (**a**) Heat map showing fragment analysis results from animals challenged with either NTHi 723 *modA2*(22)ON or *modA2*(24)OFF. The time of sampling is indicated at the top of the figure by days after initial infection, for example, day 0=initial inoculum, day 4=4 days after initial infection and so on. All fragment analysis data are presented in Supplementary Table 4; (**b**) middle ear exudate (MEE) bacterial CFU mean counts from animals infected with *modA2*(22)ON and *modA2*(24)OFF. Error bars represent s.d.; and (**c**) survival of animals infected with *modA2*(22)ON and *modA2*(24)OFF.

The ON/OFF status of the repeat tracts in both the *modA2*(22)ON and *modA2*(24)OFF infected cohorts from the starting inoculum (day 0) and subsequent days 4, 7, 10, 14, 18 and 22 after challenge were verified by fragment analysis (Fig. 5a; full data set presented in Supplementary Table 4). No gross difference in colony-forming units (c.f.u.) number or mortality was observed (Fig. 5b,c). Figure 5a shows a heat map of the *modA2*ON status of the inoculum, and the bacteria isolated from both middle ears. Animals challenged with the predominantly *modA2*ON variant (green) remained ON, whereas those animals challenged with the predominantly *modA2*OFF variant (red) showed a consistent switch from the OFF to the ON state during the course of the experimental OM study (Fig. 5a). This observation suggests that selection for *modA2*ON over *modA2*OFF occurred during overt infection of the middle ear.

### *modA2*ON forms more robust biofilms *in vitro* than *modA2*OFF

**Figure 6:**
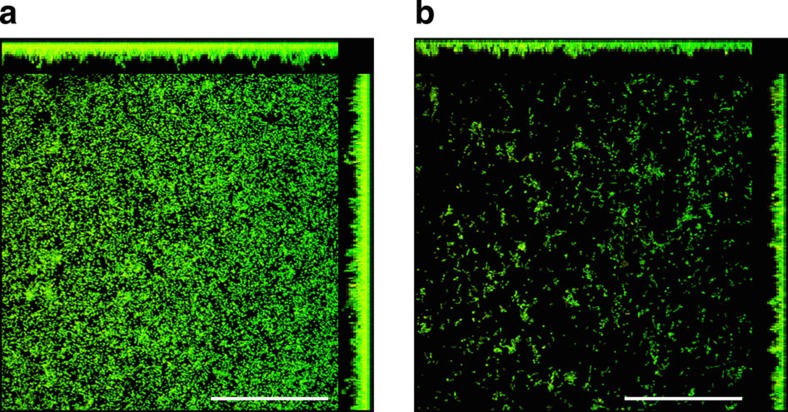
Biofilm formation using *modA2*ON and OFF strains. (**a**) Shows biofilm formation using *modA2*(22)ON. (**b**) Shows biofilm formation using *modA2*(24)OFF after 24 h growth on chambered coverglass and visualized using confocal microscopy following LIVE/DEAD BacLight staining. Scale bar shown represents 50 μm.

Given the selection for *modA2*ON in the chinchilla middle ear and the importance of biofilm formation in chronicity and recurrence of OM, we assayed these ON and OFF variants for relative ability to form a biofilm *in vitro*. Chamberslides were inoculated with either NTHI strain 723 *modA2*(22)ON or modA2(24)OFF and allowed to form biofilms for 24 h before being stained with a viable bacterial stain and subjected to confocal imaging. As shown in Fig. 6, biofilms formed by the *modA2*ON variant were notably more robust than those formed by *modA2*OFF.

## Discussion

Phenotypic analysis of all *modA* ON/OFF pairs showed that all phasevarions influence the protein profile of the outer membrane, with gross phenotypic changes evident with the five strain pairs on silver-stained gels (Fig. 3). Our in-depth analysis of multiple current vaccine candidates^50^ through western blotting, and iTRAQ quantitative mass-spectrometry of OMPs, showed that *modA* phase variation influenced the expression of several of these proteins, with some current vaccine candidates^50^ differentially expressed in multiple phasevarions (HMW1/2A, OMP P6). Most compelling in this analysis was the expression and phenotypic differences related to HMW1/2A. The HMW protein is a well-characterized adhesin expressed by NTHi, and has been investigated as a potential vaccine candidate^33^,^34^. However, our findings suggest that differential expression is occurring in three separate *modA* phasevarions. This influence appears to be direct (*modA4* and *modA5* directly affect HMW-A expression) or indirect (*modA2* ON cells show higher expression of the accessory protein HMW2B). Although HMW1/2A is known to be phase variable itself, through heptanucleotide repeats in its promoter region^37^, sequencing of HMW in all the three strain pairs show that phase-variable expression of HMW1/2A itself is likely not responsible for the large differences in expression demonstrated by western blot and ELISA in the *modA2, modA4* and *modA5* ON/OFF strain pairs. The difference in HMW expression mediated by the *modA4* phasevarion is sufficient to result in marked differences in the rate of HMW-specific opsonophagocytic killing (Fig. 4). This would likely result in selection for a *modA4*OFF subpopulation that express lower amounts of HMW. HMW has adhesion functions^51^ after selection has been relaxed; therefore, selection for adherent cells may counter select for the subpopulation of *modA4*OFF.

In this study, iTRAQ proteomic analysis of the OMP profile, and detailed gene expression studies of NTHi strain 723, containing *modA2*, the most prevalent *modA* allele found in all the clinical isolates (Fig. 1), showed that ModA2 has a major effect on gene expression and phenotype. Together with animal studies showing consistent selection for cells that have switched from *modA2*OFF to ON in our chinchilla model system of OM, this indicates that switching of the *modA2* phasevarion plays an important role in niche adaptation to the middle ear. This could be due, in part, to increased HMW expression, despite this making the cells more susceptible to an adaptive immune response. Here, the balance between adherence to host cells, a key step in colonization, and expression of this immunogenic surface protein appears to have been achieved: a *modA2*ON strain (increased in HMW1/2A expression) may be more adherent when compared with *modA2*OFF (decreased HMW1/2A). Perhaps the advantage of increased adhesion early in infection through increased HMW1/2A expression gives a selective advantage, with a gradual decrease in expression of HMW1/2A occurring over prolonged periods of host colonization, thus reducing the potential for an adaptive immune response to HMW1/2A^52^.

Expression of iron and haem-acquisition-associated genes have previously been shown to increase in NTHi recovered from middle ear effusions in patients with OM^53^. Thus, strains lacking a functional ModA2 protein (*modA2*OFF and *modA2::kan*) appear to be primed to cope well in the iron-restricted host environment through increased production of a number of iron-acquisition factors. However, this may, in fact, be counterproductive: high iron concentrations are known to increase oxidative stress through the Fenton reaction^54^, and may, in fact, decrease the fitness of *modA2*OFF strains in some host environments. Several genes involved in anaerobic metabolism are upregulated in *modA2::kan* (Table 3). Dimethylsulfoxide reductase is a virulence factor in the *Pasteurellaceae*^55^; and phosphoenolpyruvate carboxykinase, which feeds phosphoenolpyruvate into gluconeogenesis to generate a pool of glucose phosphate, is key to the pathogenesis of *Staphylococcus aureus*^56^. Several gene-encoding enzymes involved in glycogen synthesis are also upregulated—glycogen is important for biofilm formation in *Salmonella enteritidis*^57^ and *E. coli*^58^. However, many of these genes were shown to be key when studying mutants in these systems: an all or nothing approach. The subtle changes resulting from *modA2* phase variation (for example, phosphoenolpyruvate carboxykinase is only ∼1.7-fold upregulated in *modA2::kan*; enzymes involved in glycogen synthesis are all ∼1.5-fold upregulated in *modA2::kan*) appear to give no competitive advantage to *modA2*OFF compared with *modA2*ON when infecting the middle ear. Indeed, the changes in *modA2*ON are, in fact, preferentially selected. Whether this is due to the increased adhesion afforded by increased HMW-A proteins in *modA2*ON, the potential for increased oxidative stress through increased iron accumulation in *modA2*OFF leading to decreased fitness, some result of metabolic flux due to differences in the levels of respiratory and associated enzymes, or a combination of all these factors, remains to be elucidated. Moreover, as the NTHi 723 *modA2*ON strain was able to form more robust biofilms *in vitro*, this may also contribute to selection for the ON variant in the middle ear niche *in vivo*. Thus, selection against *modA2*OFF, and selection for *modA2*ON, may combine *in vivo*.

Recent work^17^,^59^,^60^ supports a key role for phase variation of individual factors in niche adaptation, independent of the established role of phase variation in immunoevasion. In this study, we have focused on the biphasic epigenetic switch that results in pleiotropic differential regulation of a phase-variable regulon of genes—the phasevarion. Previous studies have suggested a role for phasevarions in virulence^6^,^7^,^9^,^10^,^16^,^41^, but the impact of this novel biphasic epigenetic switch was untested *in vivo*. In this study, we observed for the first time a consistent selection for individuals that switched from *modA2*OFF to ON within the middle ear niche in the chinchilla model of OM. This *in vivo* data reveals a clear fitness advantage for *modA2*ON in this niche. Furthermore, we have shown that five phase-variable *modA* alleles predominate in clinical OM isolates and healthy carriers, suggesting a link between NTHi phasevarions and the potential to transmit and cause disease. All candidate phasevarions examined were shown to regulate multiple genes, including potential vaccine candidates. Defining the stable immunological target that NTHi represents requires a full analysis of the impact of phasevarions on NTHi gene expression, and future vaccine candidates will need to be assessed to confirm that their expression is not influenced by the epigenetic changes that result from phasevarion ON/OFF switching. Our recent studies describing a distinct, six-phase epigenetic switch in the major Gram-positive pathogen *Streptococcus pneumoniae*^61^ indicates that bacterial epigenetics is a key emerging field in bacterial pathogenesis and a new challenge to vaccine development for these important human pathogens.

## Methods

### Bacterial strains and cultures

NTHi strains 723, 477 and 1209 were received from the Finnish Otitis Media study group^62^. NTHi strain C486 was isolated from a child with otitis media^63^. Strain R2866 was isolated from a child with sepsis^64^. NTHi were routinely cultured in BHI broth (Oxoid) supplemented (sBHI) with hemin (1% v/v) and NAD (2 μg ml^−1^) or sBHI agar (as broth but with 1% w/v bacteriological agar; Oxoid). Liquid cultures were grown aerobically at 37 °C with shaking at 90 r.p.m. Plates were grown at 37 °C supplemented with 5% (v/v) CO_2_. *E. coli* DH5α (Coli Genetic Stock Centre, Yale University, USA) and BL21(DE3) (Merck Millipore) strains were grown at 37 °C in Luria-Bertani (LB) broth supplemented with ampicillin (100 μg ml^−1^) or kanamycin (50 μg ml^−1^) as required.

### Molecular biology

All restriction endonucleases were purchased from New England Biolabs. Primers were purchased from Sigma-Aldrich and are detailed in Supplementary Table 5. PCR was carried out as recommended by the manufacturer’s instructions (Promega; EMD Millipore, USA). Sequencing was carried out using Big Dye 3.1 (Perkin Elmer) and PCR products purified using the Qiagen PCR purification kit according to the manufacturer’s instructions. Samples were sequenced by the Griffith University DNA sequencing facility (GUDSF), Brisbane, Australia, or the Australian Equine Genetics Research Facility, University of Queensland, Brisbane. Primers used for sequencing the HMW promoter region (HMW1-F; HMW2-F; HMW3-F and HMW-R; Supplementary Table 5) were based on those used previously^37^. The *modA* DRD region was analysed as previously described^6^. Briefly, PCR products encompassing the DRD were amplified using primers Him6 and Him11 (Supplementary Table 5), sequenced and compared with *modA* allele reference sequences described^6^,^15^. Natural ON and OFF strains were isolated by fragment length analysis of the *modA* repeat tract of multiple single colonies using the fluorescently labelled forward primer Him1F and the reverse primer Him3 (Supplementary Table 5)^6^, and fragments were analysed by AEGRC or GUDSF. Strains containing >90% ON or OFF were considered to be natural ON or OFF, respectively, and were used in subsequent studies.

PCR products generated for cloning into the pET51 Ek/LIC cloning vector (EMD Millipore) were prepared using KOD Hot-start DNA polymerase (EMD Millipore) using gene-specific primers for *modA2* or *siaB,* (Supplementary Table 5) according to the manufacturer’s instructions. Overexpression of ModA2 and SiaB was carried out using *E. coli* BL21 cells, which were induced by the addition of IPTG to a final concentration of 0.5 mM for 2 h at 37 °C with shaking at 120 r.p.m.

### RNA extraction

Triplicate cultures of NTHi strains 723 *modA2*ON and 723 *modA2::kan* were grown to exponential phase (optical density at 600 nm=0.3 to 0.4) in sBHI broth before RNA extraction. Growth rates of strain pairs used to make RNA for microarray comparison were equivalent, ensuring that the samples taken were in the same growth phase. Culture media for RNA preps were free of antibiotics. Approximately 100 μg of total RNA was prepared from each sample using the RNeasy Midi Kit according to the manufacturer’s instructions (Qiagen). The triplicate samples were pooled and the integrity and concentration of RNA was determined via micro-fluidic analysis on a bio-analyser (Agilent Technologies).

### Microarray analysis

All the microarray analyses were performed on *H. influenzae* genome arrays (TIGR; http://pfgrc.tigr.org/). Each microarray consists of 4,454 70-mer oligonucleotides representing ORFs from *H. influenzae* strains Rd KW20, 86-028NP, R2846 and R2866. Methods and analysis were as previously described^65^. Briefly, 5 μg of each total RNA sample was labelled using random hexamers and direct incorporation of fluorescently Cy3- or Cy5-labelled nucleotides as described^66^. Hybridizations were performed in triplicate and incorporated a dye swap to account for dye bias. After hybridization, arrays were washed and scanned on an Agilent G2565BA microaray scanner. Images of the hybridizations were analysed using Imagene 5.5 (BioDiscovery) and the mean foreground, mean background and spot/signal quality determined. Primary data were imported into an in-house installation of BASE (http://kidney.scgap.org/base). After print-tip intensity-independent Lowess normalization, differential expression was defined using a robust statistical method rather than simple fold change. All the genes were ranked using the B-statistic method where both fold change and variance of signals in replicates are used to determine the likelihood that genes are truly differentially expressed.

### Quantitative real-time PCR

Oligonucleotides (Supplementary Table 5) were designed using Primer Express 1.0 software (ABI Prism; PE Biosystems). Real-time PCR reactions were performed in triplicate using RNA isolated from 723 *modA2*ON and 723 *modA2*OFF. cDNA was synthesized using NEB Protoscript II and random hexamers (Invitrogen; 50 ng μl^−1^) according to the manufacturer’s instructions. Reverse transcriptase reactions lacking Protoscript II were performed as a negative control. All the real-time PCR reactions were performed in a 25-μl mixture containing a 1 in 5 dilution of the cDNA preparation (5 μl), 10xSYBR Green buffer (PE Applied Biosystems) and 2 μM of each primer. 16S RNA was used as the standard control in each quantitative PCR. Amplification and detection of specific products were performed with the ABI Prism 7700 sequence-detection system (PE Applied Biosystems) with the following cycle profile: 95 °C for 10 min, followed by 45 cycles of 95 °C for 15 s and 60 °C for 1 min. The data were analysed with ABI prism 7700 (version 1.7) analysis software. Relative gene expression between 723 *modA2*OFF and 723 *modA2*ON was determined using the 2^−ΔΔCT^ relative quantification method.

### SMRT sequencing and methylome analysis

Genomic DNA from each natural *modA*ON and kanamycin knockout pair was prepared using the Qiagen genomic DNA midi kit according to the manufacturer’s instructions. SMRT and methylome analysis was carried out as done previously^21^,^22^. Briefly, genomic DNA was sheared to an average length of ∼10 kb using g-TUBEs (Covaris, Woburn, MA, USA) and SMRTbell template-sequencing libraries were prepared using sheared DNA. DNA was end repaired, then ligated to hairpin adaptors. Incompletely formed SMRTbell templates were degraded with a combination of Exonuclease III (New England Biolabs; Ipswich, MA, USA) and Exonuclease VII (USB; Cleveland, OH, USA). Primer was annealed and samples were sequenced on the PacBio RS II (Menlo Park, CA, USA) using standard protocols for long insert libraries. Plasmid midi-preps from *E. coli* cells expressing NTHi 723 ModA2 and a negative control expressing a non-methylase (SiaB), were prepared using the Qiagen plasmid midi kit according to the manufacturer’s instructions, and analysed as above.

### Preparation of OMPs from NTHi

NTHi *modA* ON/OFF pairs were grown in sBHI broth (50 ml) at 37 °C overnight with shaking at 100 r.p.m. The cells were pelleted at 4,500 r.p.m. for 15 min at 4 °C, resuspended in 4 ml 10 mM HEPES-NaOH pH7.5 and OMPs were prepared as detailed previously^67^. Briefly, cells were lysed by sonication, and debris pelleted as above. Sarcosyl was added to the clarified supernatant to a final concentration of 1% and incubated at 25 °C for 30 min. Supernatants were then centrifuged at 110,000*g* for 90 min. Pellets were resuspended in 10 mM HEPES-NaOH pH7.5, sarcosyl added to a final concentration of 1%, and incubation and centrifugation steps repeated twice more. Final pellets containing the OMP-enriched fraction were resuspended in 100 μl of 10 mM HEPES-NaOH pH7.5 and the protein concentration quantified using the BCA protein assay kit according to the manufacturer’s instructions (Thermo Scientific).

### Western blot analysis

Each of the OMP preparations (5 μg) were run on the Novex Bis-Tris pre-cast gel system with MOPS running buffer according to the manufacturer’s instructions (Life Technologies). Ammoniacal silver staining was carried out to visualize proteins. Western blotting was carried out using nitrocellulose membranes (Bio-Rad) and standard protocols^68^. All mouse (AD6 anti-HMW1/2A^34^; 1F4 anti-Hia^35^) and rabbit (LB1 anti-OMP P5 (ref. 28); chimV4 anti-OMP P5; anti-OMP P5) primary antibodies were used at a dilution of 1:2,500; all chinchilla (anti-OMP P2; anti-LPD^27^; anti-PDM^27^; anti-OMP P5/P6) primary antibodies at a dilution of 1:250. Anti-mouse-AP and anti-rabbit-AP secondary antibodies were used at a dilution of 1:5,000 (Sigma-Aldrich); protein A-AP secondary antibody was used at a dilution of 1:500 (Sigma-Aldrich) in blots where chinchilla primary antibodies were used. Blots were developed using SigmaFAST NBT/BCIP tablets according to the manufacturer’s instructions (Sigma-Aldrich). All the primary antibodies were raised by the authors laboratories, with specific references for those described previously.

### ELISA assay

ELISA assays were carried out using standard protocols^68^ in 96-well Maxisorb plates (NUNC; Thermo Scientific). Cells were diluted to a 0*D*_600_ of 0.2 (2 × 10^8^ c.f.u. ml^−1^), with 50 μl added per well. All the strains were assayed in triplicate. Primary antibody AD6 against HMW^34^ was used at a starting concentration of 1:200 (NTHi strains C486 and 477) or 1:10,000 (NTHi strain 723) and serially diluted two-fold in 1 × PBS pH 7.9. Secondary antibody (goat anti-mouse HRP conjugate; Sigma-Aldrich) was used at a concentration of 1:10,000. Antibody was detected using TMB single-substrate solution as recommended by the manufacturer (Sigma-Aldrich). The data were plotted as antibody dilution (*x* axis) versus absorbance at 450 nm (*y* axis), and data from specific titres in the linear range of the response curve picked for statistical analysis.

### iTRAQ analysis

iTRAQ 1D nanoLC ESI MS/MS was carried out by the Australian Proteome Analysis Facility (APAF), Macquarie University, Sydney, Australia. Approximately 10 μg of OMP preparation from duplicate samples of each *modA* ON/OFF pair was supplied for the analysis. Samples were buffer exchanged into 0.25 M TEAB and 0.05% SDS and quantified samples were reduced with TCEP, alkylated with MMTS and digested with trypsin. Digested samples were labelled, passed through an SDS removal column (Thermo Fisher) and dried. Labelled samples were resuspended in 50 μl of loading/desalting solution (0.1% formic acid and 2% acetonitrile 97.9% water). Sample (20 μl) was injected onto a peptide trap (Michrome peptide Captrap) for pre-concentration and desalted with 0.1% formic acid, 2% acetonitrile. Peptides were eluted from the column using a linear solvent gradient, with steps, from mobile phase A: mobile phase B (98:2) to mobile phase A: mobile phase B (65:35) where mobile phase A is 0.1% formic acid and mobile phase B is 90% ACN/0.1% formic acid at 600 nl min^−1^ over a 100-min period. After peptide elution, the column was cleaned with 95% buffer B for 15 min and then equilibrated with buffer A for 25 min before next sample injection. The reverse phase nanoLC eluent was subject to positive ion nanoflow electrospray analysis in an information dependent acquisition mode (IDA). In IDA mode, a TOFMS survey scan was acquired (*m*/*z* 400–1,500, 0.25 s), with the 10 most intense multiply charged ions (counts >150) in the survey scan sequentially subjected to MS/MS analysis. MS/MS spectra were accumulated for 200 ms in the mass range *m*/*z* 100–1,500 with the total cycle time of 2.3 s. The experimental nanoLC ESI ms/ms data were submitted to ProteinPilot V4.2b (AB Sciex) for data processing.

### MIC assay

The MIC was measured by broth microdilution in triplicate experiments as described previously^69^ using mid-log phase NTHi cells grown aerobically in sBHI. Briefly, 50 μl of each culture was added to 96-well plates in which antibiotics had been serially diluted, and plates grown at 37 °C with 5% CO_2_ for 24 h. The MIC (mg l^−1^) was determined as the last dilution at which turbidity was observed following overnight growth. All the assays were performed in triplicate.

### Opsonophagocytic killing assays

The growth conditions of the bacteria, the growth and differentiation of the HL-60 cells, (ATCC CCL-240) and the opsonophagocytic assay itself were performed as described previously^33^,^42^. The opsonophagocytic assay was performed in 5-ml capped polystyrene tubes (Sarstedt, Newton, NC). The complement source was human serum collected from a single healthy adult that was adsorbed to remove serum IgG by passing aliquots repeatedly over a protein G affinity column at 4 °C. Antibody (guinea pig anti-strain 12 HMW1/HMW2 antiserum^42^) was serially diluted, and ∼5 × 10^3^ c.f.u. mid-log phase bacterial cells were added to each dilution, and tubes incubated at 37 °C with 5% CO_2_ for 15 min. Following this, complement was added, followed by immediate addition of differentiated HL-60 cells. Tubes were incubated at 37 °C with 200 r.p.m. horizontal shaking. At the end of the 90-min incubation period, c.f.u. were calculated by plating 10 μl of each culture and incubating overnight at 37 °C with 5% CO_2_. The per cent killing at each serum dilution was calculated by determining the ratio of the bacterial colony count at each dilution to that of the complement control using the *modA4* ON/OFF pair (NTHi strain C486). Pre-immune sera and anti-Hia sera were used as negative controls.

### Biofilm formation on chambered coverglass slides

Formation of NTHI biofilms was performed in eight-well-chambered coverglass slides (Thermo Scientific, Waltham, MA) as described previously^70^. Briefly, mid-log phase cultures of NTHi strain 723 *modA2*ON and *modA2*OFF grown in sBHI were diluted with fresh pre-warmed media and used to inoculate 4 × 10^4^ c.f.u. in 200 μl total volume per well. Slides were incubated at 37 °C with 5% atmospheric CO_2_ and the growth medium was replaced with fresh medium after 16 h. Twenty-four hours after seeding, biofilms were stained with LIVE/DEAD BacLight stain (Life Technologies) and fixed overnight in fixative (1.6% paraformaldehyde, 2.5% glutaraldehyde, 4% acetic acid in 0.1 M phosphate buffer, pH 7.4). Fixative was replaced with saline before imaging on a Zeiss 510 Meta-laser scanning confocal microscope; images were rendered with Zeiss Zen software.

### Chinchilla model of NTHi-induced OM

Adult chinchillas (*Chinchilla lanigera*) were supplied by Rauscher’s Chinchilla Ranch, LaRue, OH. Chinchillas were not sex-differentiated, and were classed as adult if weighing between 500–700 grams. Chinchillas were allowed to acclimate in the vivarium for 7–10 days before beginning the study. In two separate studies, cohorts of five chinchillas each were established, then challenged intranasally and transbullarly with either: (1) NTHi strain 723 *modA2*(22)ON or (2) NTHi strain 723 *modA2*(24)OFF at a challenge dose of ∼1 × 10^8^ c.f.u. intranasally and 750 c.f.u. transbullarly. A total of 10 animals (20 ears) were challenged with each of these variants of NTHI strain 723. The animals were then monitored daily via video otoscopy for 22 days. Delivered doses were confirmed by dilution plate counts of inocula on chocolate agar. On days 2, 4, 7, 10, 14, 18 and 22 after challenge epitympanic taps (removal of a small aliquot of fluid from the middle ear space) were performed as described previously. On collection of samples, 10 μl were placed in a sterile Eppendorf tube on ice. This aliquot was used for evaluation of the colony-forming unit of NTHi per ml sample. The remainder of the collected samples was immediately centrifuged at 4 °C for 3 min, the supernatant was carefully removed and the pellet was snap frozen in liquid nitrogen and stored at −80 °C. On day 22 (unless removed from the study early owing to morbidity), anaesthetized chinchillas were euthanized and the bullae were dissected from the skull. The left bulla from each chinchilla was aseptically opened and imaged. The middle ear mucosa, including any adherent mucosal biofilm was removed, homogenized in sterile PBS and snap frozen.

With regard to animal treatment and handling, all protocols were approved by the Nationwide Children’s Hospital Animal Care and Use Committee, in accordance with the US Department of Health and Human Services Guide for the Care and Use of Laboratory Animals.

### Fragment analysis to determine *modA2* ON/OFF status in chinchilla samples

Fluid samples were taken from the left and right middle ears on days 0, 2, 4, 7, 10, 14, 18 and 22 from each cohort of 10 chinchillas that had been challenged with either strain 723 *modA2*(22)ON or strain 723 *modA2*(24)OFF. The ratio of *modA2*ON and *modA2*OFF from the starting inoculum (day 0) compared with days 4, 7, 10, 14, 18 and 22 was verified via fragment analysis for each of the chinchillas. Samples were thawed briefly on ice, with 1 μl serving as the template in a 25 μl GoTaq PCR reaction (Promega) using primers Him1F and Him3 (Supplementary Table 5) as described previously^6^. Samples were run using GeneScan fluorescent-PCR fragment-length analysis by GUDSF, and analysed using Peak Scanner software (Applied Biosystems).

## Additional information

**How to cite this article:** Atack, J. M. *et al.* A biphasic epigenetic switch controls immunoevasion, virulence and niche adaptation in non-typeable *Haemophilus influenzae*. *Nat. Commun.* 6:7828 doi: 10.1038/ncomms8828 (2015).

## Supplementary Material

Supplementary Information Supplementary Figures 1-5, Supplementary Tables 1-5 and Supplementary References

Supplementary Data 1 HMW protein expression (measured by ELISA) from *modA2, modA4* and *modA5* ON/OFF strain pairs. Specific titres from the linear range of the response curve used to calculate statistical significance are highlighted by a black box.

Supplementary Data 2 iTRAQ data – data are presented as raw files received from the Australian Proteome Analysis Facility (APAF), with all identified hits presented in the data file. Green cells indicate the protein is down-regulated ON:OFF; pink cells indicate the protein is upregulated ON:OFF. Yellow cells indicate a statistically significant difference as judged by Stouffer's P-value and Stouffer's adjusted value. Several proteins for each strain showed >1.5 or <0.65 differences in regulation ON:OFF, but these are not included in Table 2 as they are either not OMPs, or they themselves are phase variable, and we cannot be certain that *modA* phase variation results in the differential expression of these latter proteins.

## Figures and Tables

**Table 1:** Summary of SMRT sequencing and methylome analysis of representative strains containing the five *modA* alleles under study.

***modA*** **allele**	**NTHi strain**	**Clinical symptoms**	**Accession number**	**Methylation sequence**	**Systematic name**	**Number of sites in genome**	**Genome size (bp)**	**Predicted ORFs**
*modA2*	723	OM	CP007472	5'-CCGA(^m6^A)-3'	M.Hin723I	2,270	1,887,620	1,868
*modA4*	C486	OM	CP007471	5'-CG(^m6^A)G-3'	M.HinC486I	6,203	1,846,507	1,783
*modA5*	477	OM	CP007470	5'-AC(^m6^A)GC-3'	M.Hin477I	2,548	1,846,259	1,813
*modA9*	1209	OM	JMQP01000000	5'-CCTG(^m6^A)-3'	M.Hin1209I	2,504	1,895,979	2,247
*modA10*	R2866	Blood	CP002277*	5'-CCT(^m6^A)C-3'	M.Hin2866I	1,244	1,932,238	1,905

^*^Strain already annotated and submitted (October 2010) to the EMBL/GenBank/DDBJ databases. A full summary of SMRT sequencing/methylome analysis derived data is presented in Supplementary Fig. 1.

**Table 2:** iTRAQ quantitative mass spectrometry analysis using OMPs from each ON/OFF pair.

**Gene ID**	**Gene**	**Ratio ON:OFF** *	**Stouffers** ***P***-**value**	**Stouffer Adj**
*Increased expression in 723 modA2 ON*				
723_01555	*N*-acetylneuraminate epimerase NanM	2.36	5.40E−14	6.32E−13
723_01788	Adhesin translocation protein HMW2B	2.05	0	0
				
*Decreased expression in 723 modA2 ON*				
723_01435	Haem/haemopexin utilization protein C HxuC1	0.28	0	0
723_00620	Transferrin-binding protein 1	0.32	4.74E−08	3.45E−07
723_01615	Major ferric iron-binding protein HitA†	0.37	0	0
723_01434	Haem/haemopexin transporter protein HxuB†	0.41	0	0
723_01596	Haemin receptor, HemR	0.42	0	0
723_01306	15 kDa peptidoglycan-associated lipoprotein OMP P6	0.6	2.57E−04	1.12E−03
				
*Increased expression in C486 modA4 ON*				
C486_00892	OMP P2	1.52	0	0
				
*Increased expression in 477 modA5 ON*				
477_00572	OMP P5	1.65	0	0
				
*Increased expression in R2866 modA10 ON*				
R2866_0725	Adhesin Hia‡§	11.53	0	0
R2866_0192	OMP P6	1.85	0.046	0.17
R2866_1237	OMP P5	1.65	7.39E−03	0.043

Gene designations from analysis with our SMRT produced genomes for each strain; differences are represented as ON:OFF; statistical significance is measured using Stouffers *P* value and Stouffers adjusted (Adj) value, as the results are the interpretation and comparison of two independently prepared sets of OMPs from each ON/OFF pair. Schematics of differentially regulated genes with locations of ModA methylation motifs are depicted in Supplementary Fig. 3.

^*^Only samples with an ON:OFF ratio >1.5 or <0.7 were included. Complete iTRAQ data for all the five strain pairs are presented as Supplementary Data 2.

^†^Indicates identification by microarray and iTRAQ.

^‡^Identified by western blotting and iTRAQ.

^§^Hia was subsequently shown to be regulated in a *modA10*-independent manner.

**Table 3:** Differentially expressed genes in the NTHi strain 723 *modA2*ON microarray.

**Gene**	**Microarray ID**	**Description**	**Ratio**	**qRT-PCR**	**B-Stat**
*Reduced expression in the H. influenzae strain 723 modA2 mutant*					
723_01712	NTHI0007	Formate dehydrogenase major subunit *fdxG*	−1.50		2.77
723_01520	Hflu203001461	Alcohol dehydrogenase, class III	−1.53		2.42
723_01710	NTHI0010	Formate dehydrogenase, iron-sulfur subunit *fdxH*	−1.53		1.66
723_00429	Hflu103001790	L-lactate permease *lctP*	−1.79		3.08
723_00946	Hflu203000185	DL-methionine transporter ATP-binding subunit *metN*	−1.96	−3.56±0.741*	4.80
					
*Increased expression in the H. influenzae strain 723 modA2 mutant*					
723_01433	ORF02024	Haem/haemopexin-binding precursor *hxuA*	1.45		0.86
723_00220	NTHI1806	Glycogen synthase *glgA*	1.49		1.84
723_00222	NTHI1808	Glycogen debranching enzyme *glgX*	1.52		1.27
723_01160	HI0534	Aspartate ammonia-lyase *aspA*	1.52		2.66
723_00775	ORF01899	Fumarate reductase subunit D *frdD*	1.56		0.89
723_01524	HI0181	Formate transporter *focA*	1.57		1.14
723_01327	Hflu203001649	Iron (chelated) ABC transporter *yfeB*	1.61		1.32
723_00221	NTHI1807	Glucose-1-phosphate adenylyltransferase *glgC*	1.61		3.76
723_00223	NTHI1809	Glycogen branching enzyme *glgB*	1.66		2.21
723_00419	HI0809	Phosphoenolpyruvate carboxykinase *pckA*	1.69		1.00
723_01614	ORF00565	Iron (III) ABC transporter, permease protein *hitB*	1.75		4.39
723_01328	Hflu203001648	Iron chelated ABC transporter permease *yfeC*	1.90		1.69
723_01329	Hflu203001647	Iron chelated ABC transporter permease *yfeD*	2.08		3.98
723_00591	Hflu203000516	Anaerobic DMSO chain C *dmsC*	2.09		1.26
723_01434	Hflu103000469	Haem/haemopexin-binding *hxuB*†	2.14		1.15
723_01326	Hflu203001650	Iron chelated ABC transporter *yfeA*	2.17	2.166±0.667*	5.83
723_00589	Hflu203000518	Anaerobic DMSO chain A *dmsA*	2.22	2.14±0.839*	2.38
723_00437	HI1210	Malate dehydrogenase *mdh*	2.28		5.50
723_00590	Hflu203000517	Anaerobic DMSO chain B *dmsB*	2.65		2.67
723_01615	Hflu203001388	Iron-utilization *hitA*†	4.04	2.08±0.397*	4.72

The genes listed are either down- or upregulated in the *H. influenzae* 723 *modA2::kan* mutant strain compared with the *modA2*ON strain. The identity of the gene is indicated with our SMRT-derived genomic annotation (accession number CP007472), the original identifier from the microarray, and a description of each gene. The average ratio presented is the mean of NTHi strain 723 *modA2::kan* mutant:723 *modA2*ON from six replicate spots on three independent microarrays, incorporating a dye swap. Only those genes with an expression value >1.4-fold were included in this table.

^*^Gene expression was confirmed by quantitative RT–PCR in NTHi strain 723 *modA2*ON and 723 *modA2*OFF strain variants.

^†^Identified by both microarray and iTRAQ. B-stat (B-statistic) represents the log-odds that the gene is differentially expressed. A threshold in the B statistic of 0.0 was adopted as genes with a B score >0 have a >50% probability of being truly differentially expressed. All the microarray data are presented in Supplementary Table 3. Schematics of differentially regulated genes with locations of ModA methylation motifs are depicted in Supplementary Fig 3.
