# Supplementary material for: A biphasic epigenetic switch controls immunoevasion, virulence and niche adaptation in non-typeable Haemophilus influenzae
Source: Nat Commun. 2015 Jul 28;6:7828. doi: 10.1038/ncomms8828 (PMC4525171; doi:10.1038/ncomms8828)
Supplement: Supplementary Information — Supplementary Figures 1-5, Supplementary Tables 1-5 and Supplementary References [file ncomms8828-s1.pdf]

Supplementary Figure 1

C486 *modA4* genome summary

a

Sample	Contigs	# of Bases
C486 *modA4* ON	1	1,846,507
C486 *modA4*::kan	1	1,847,851

b

Methyltransferase Specificity	Modified Base	*modA4* ON	*modA4* ::kan
5′-GATC-3′<br>3′-CTAG-5′	m6A	100%	99.9%
5′-CGAG-3′<br>3′-GCTC-5′	m6A	99.2%	0.1%
5′-ACAN_6RTTG-3′<br>3′-TGTN_6YAAC-5′	m6A	100%<br>98.9%	100%<br>98.5%
5′-CTKMAG-3′<br>3′-GAMKTC-5′	m6A	69.8%<br>*	67.1%<br>*
5′-VCTGGAG-3′<br>3′-BGACCTC-5′	m6A	52%<br>50%	57%<br>53%

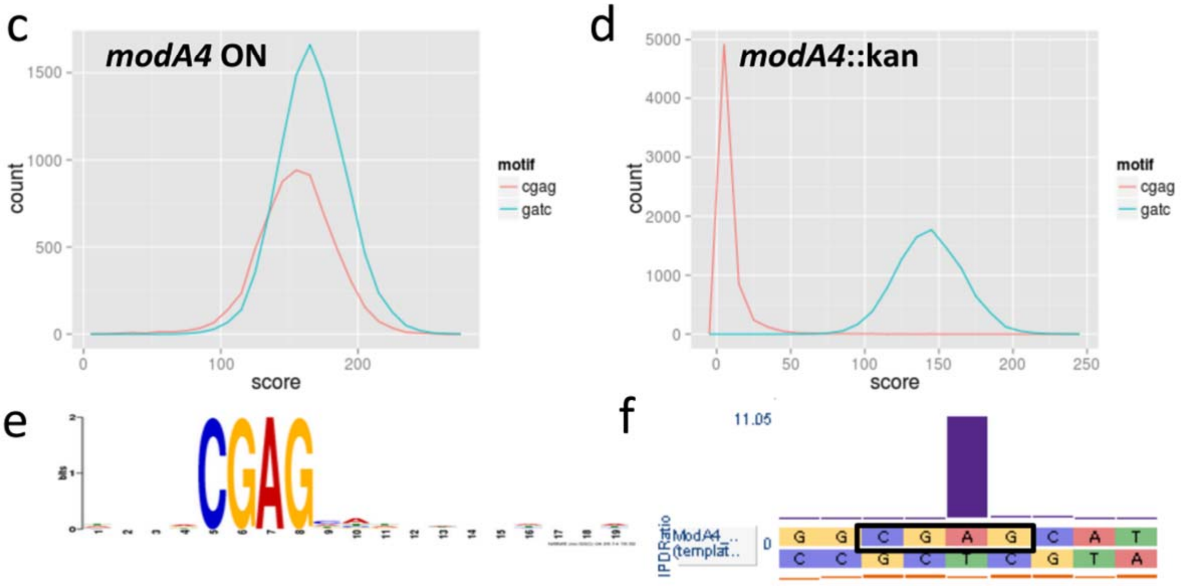

g

Motif	Total in Genome	# in Coding Region	% in Coding Region	# in non-Coding Region	% in non-Coding Region	Positive Strand	Negative Strand	Observed # Motifs	Expected # Motifs	Percent (obs/exp)
CGAG	6203	2670	43.0	3533	57.0	3129	3074	6203	7852	79.0

477 *modA5* genome summary

a

Sample	Contigs	# of Bases
477 *modA5* ON	1	1,846,259
477 *modA5*::kan	1	1,847,538

b

Methyltransferase Specificity	Modified Base	*modA5* ON	*modA5* ::kan
5′-GATC-3′<br>3′-CTAG-5′	m6A	99.0%	96.8%
5′-ACAGC-3′<br>3′-TGTCG-5′	m6A	99.8%	0%
5′-AAAYN$_6$RTAY-3′<br>3′-TTTRN$_6$YATR-5′	m6A	97.1%<br>99.1%	91.6%<br>95.6%
5′-CYTAN$_5$RTC-3′<br>3′-GRATN$_5$YAG-5′	m6A	97.8%<br>95.8%	95.1%<br>92.7%

c

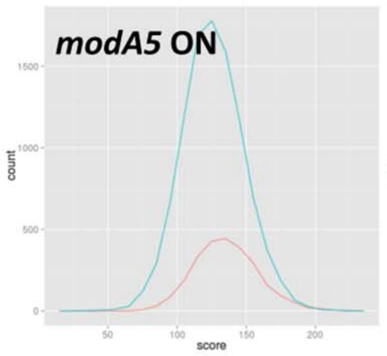

d

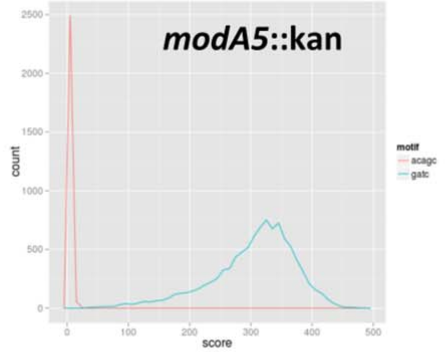

e

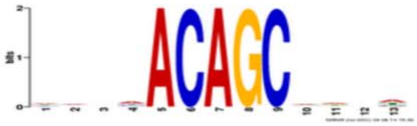

f

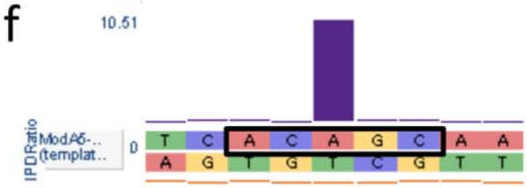

g

Motif	Total in Genome	# in Coding Region	% in Coding Region	# in non-Coding Region	% in non-Coding Region	Positive Strand	Negative Strand	Observed # Motifs	Expected # Motifs	Percent (obs/exp)
ACAGC	2548	1428	56.0	1120	44.0	1302	1246	2548	2434	104.7

1209 *modA9* genome summary

a

Sample	Contigs	# of Bases
1209 *modA9* ON	3	1,255,033<br>544,507<br>126,439
1209 *modA9*::kan	1	1,912,859

b

Methyltransferase Specificity	Modified Base	*modA9* ON	*modA9* ::kan
5′-G**A**TC-3′<br>3′-CT**A**G-5′	m6A	98.7%	99.2%
5′-GA**A**NNNNNNNTAYG-3′<br>3′-CTTNNNNNNN**A**TRC-5′	m6A	97.9%<br>99.4%	99.2%<br>99.4%
5′-G**A**GNNNNNRTC-3′<br>3′-CTCNNNNNY**A**G-5′	m6A	93.0%<br>91.7%	93.0%<br>92.0%
5′-CGWA**A**T-3′<br>3′-GCWTTA-5′	m6A	96.3%	97.0%
5′-CCTG**A**-3′<br>3′-GGACT-5′	m6A	95.6%	5.7%
5′-GCCTGC-3′<br>3′-CGGACG-5′	5mC?	6.7%	6.8%

c

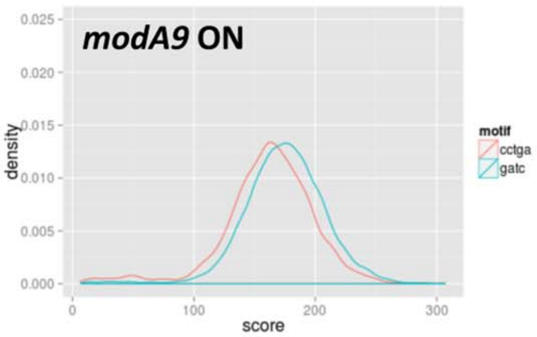

d

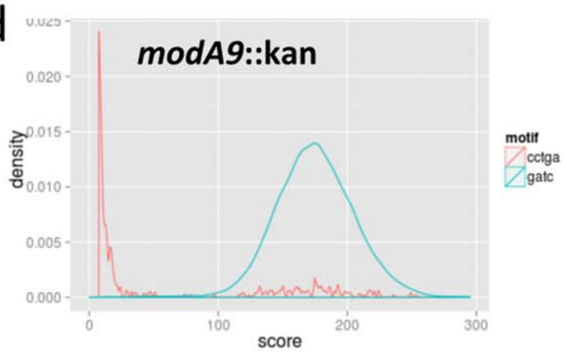

e

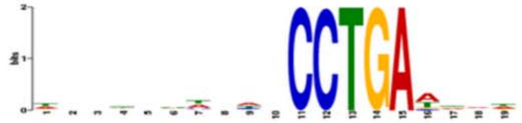

f

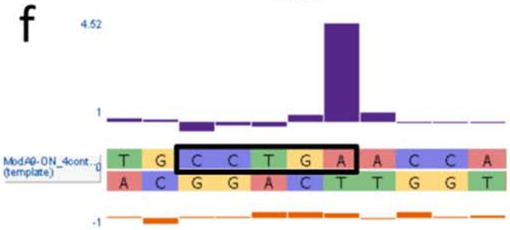

g

Motif	Total in Genome	# in Coding Region	% in Coding Region	# in non-Coding Region	% in non-Coding Region	Positive Strand	Negative Strand	Observed # Motifs	Expected # Motifs	Percent (obs/exp)
CCTGA	2503	1368	54.7	1135	45.3	1296	1207	2503	2663	94.0

R2866 *modA10* genome summary

a

Sample	Contigs	# of Bases
R2866 *modA10* ON	1	1,932,283
R2866 *modA10*::kan	1	1,933,738

b

Methyltransferase Specificity	Modified Base	*modA10* ON	*modA10* ::Kan
5′-G**A**TC-3′<br>3′-CT**A**G-5′	m6A	99.4%	99.4%
5′-CCT**A**C-3′<br>3′-GGATG-5′	m6A	97.4%	0.4%
5′-CA**A**YNNNNNNTAAA-3′<br>3′-GTTRNNNNNN**A**TTT-5′	m6A	98.5%<br>99.5%	97.7%<br>99.8%
5′-**A**CGNNNNNRAAT-3′<br>3′-TGCNNNNNYTT**A**-5′	m6A	99.9%<br>99.9%	98.9%<br>99.2%

c

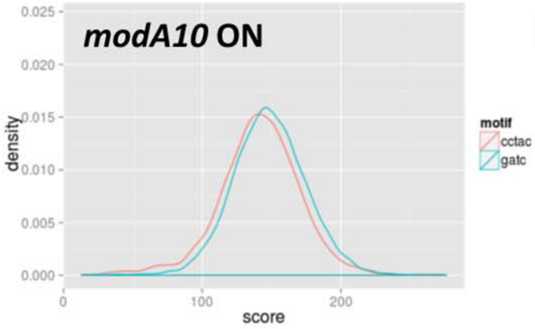

d

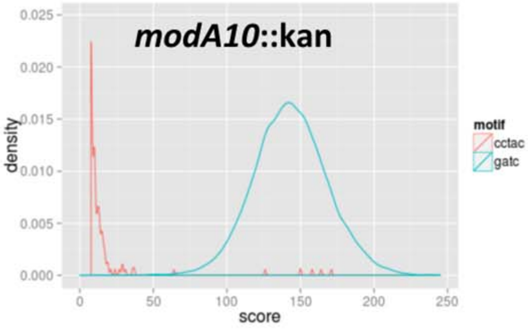

e

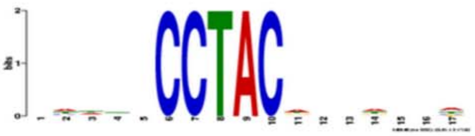

f

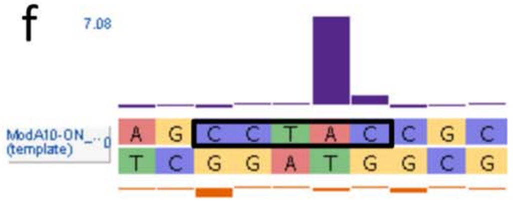

g

Motif	Total in Genome	# in Coding Region	% in Coding Region	# in non-Coding Region	% in non-Coding Region	Positive Strand	Negative Strand	Observed # Motifs	Expected # Motifs	Percent (obs/exp)
CCTAC	1244	384	30.9	860	69.1	617	627	1244	2714	45.8

723 *modA2* genomic DNA & pET51::*modA2* plasmid DNA

a

Sample	Contigs	# of Bases
723 *modA2* ON	1	1,887,620
723 *modA2*::kan	1	1,889,018

b

Methyltransferase Specificity	Modified Base	modA2 ON	modA2 Kan	modA2 OFF
5′-G**A**TC-3′<br>3′-CT**A**G-5′	m6A	99.8%	99.9%	99.8%
5′-TC**A**N$_6$TRCC-3′<br>3′-AGTN$_6$**A**YGG-5′	m6A	100%<br>100%	99.8%<br>99.8%	100%<br>99.3%
5′-**A**TGN$_6$CCT-3′<br>3′-TACN$_6$GG**A**-5′	m6A	99.6%<br>99.0%	99.6%/<br>99.0%	99.4%<br>98.6%

f

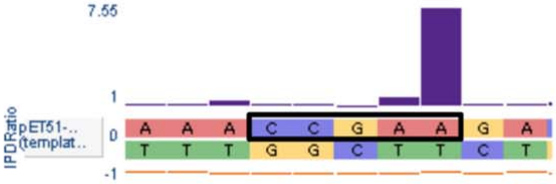

g

Motif	Total in Genome	# in Coding Region	% in Coding Region	# in non-Coding Region	% in non-Coding Region	Positive Strand	Negative Strand	Observed # Motifs	Expected # Motifs	Percent (obs/exp)
CCGAA	2270	1284	56.6	986	43.4	1130	1140	2270	2488	91.2

h

Motif	Modified Position	Modification Type	% Motifs Detected	# Of Motifs Detected	# Of Motifs In Genome	Mean Modification QV	Mean Motif Coverage	Partner Motif
GATC	2	m6A	100.00	52	52	3133.6	6774.7	GATC
CCGAA	5	m6A	100.00	19	19	2272.1	6439.1	
Not Clustered	0		4.89	713	14,587	68.7	6457.9	

Supplementary Figure 1 – Summary of SMRT genome and methylome analysis of all five *modA* ON/OFF strain pairs. Each separate strain is summarized together in a single composite image on a single page, which each panel in each summary page corresponding to the following legend - **a)** summary of the size of each SMRT sequenced genome is given for each strain pair (*modA* ON and *modA::kan*). The ON genome for strains 723, C486, 477 and 1209 was annotated and submitted to NCBI Genbank as indicated in the main text; **b)** table summarizing all the methyltransferase activities detectable in each genome, with % values in each table referring to the % methylation detected in that strain for the particular motif reported. The relevant methyltransferase signature corresponding to ModA is highlighted with a thick black box. For strain 723 (*modA2*; fifth composite panel) we also carried out SMRT sequencing of the OFF genome, and a summary of methyltransferase activities detected in this strain is also given in this table; **c)** and **d)** An example of the statistical scores in the ON **(c)** compared with the *kan* **(d)** strain for the motif assigned for that particular ModA protein compared with the dam methyltransferase (GATC), showing that methylation is detectable in the *modA::kan* strains; **e)** the sequence recognized by each ModA protein represented as a weblogo consensus sequence; **f)** a typical example of the motif in each genome with inter pulse duration (IPD) score (or plasmid vector for the ModA2 methyltransferase) shown, with the size of the IPD directly proportional to the size of the bar [1]; **g)** shows results of bioinformatics analysis of each ON genome showing statistics for the relevant ModA motif; and panel **h)**, for 723 (*modA2*) only, shows results from sequencing of plasmid vector over-expressing ModA2 – the number of ModA2 CCGAA motifs and the number of Dam GATC motifs is shown compared to the number detected as methylated.

Supplementary Figure 2

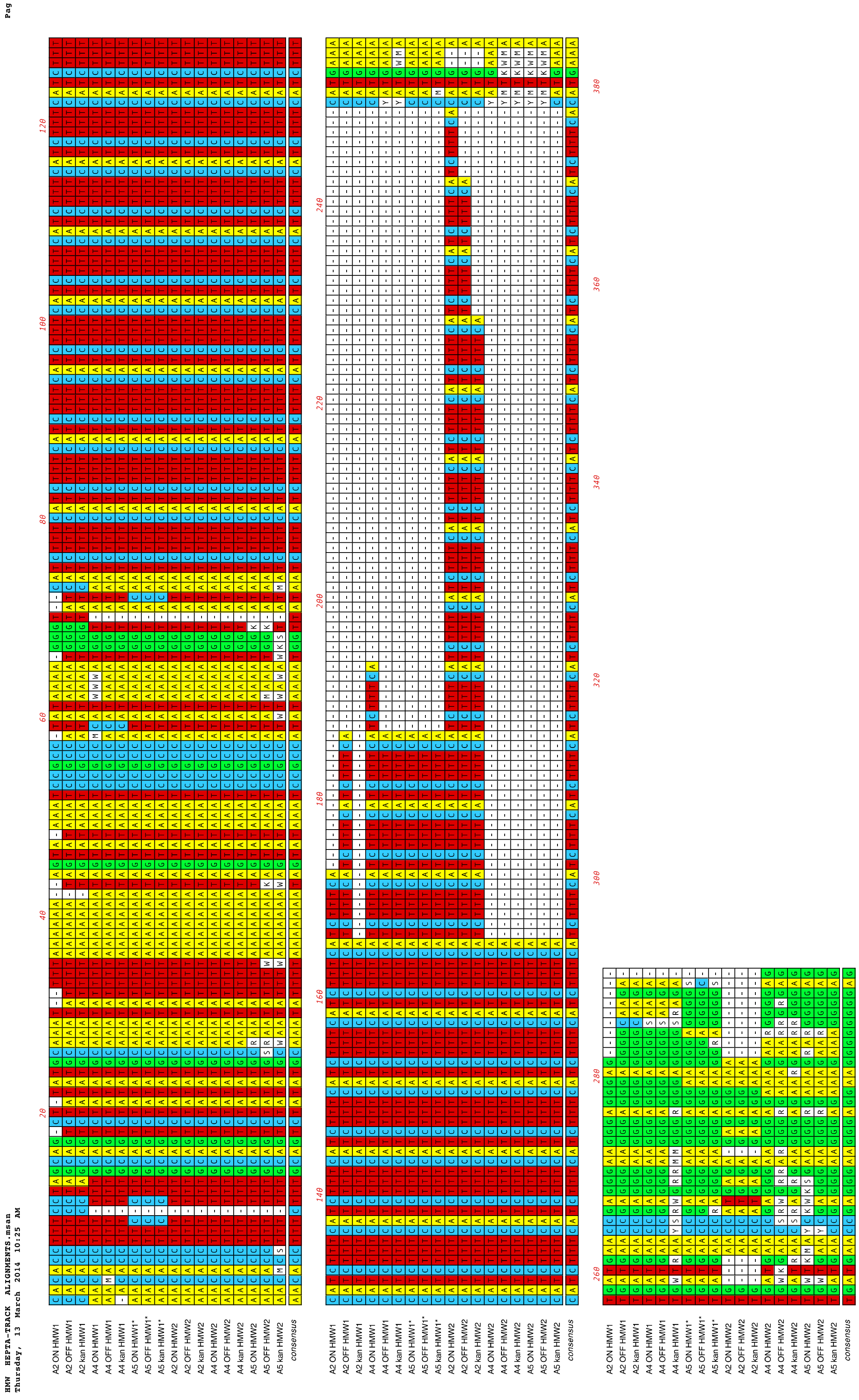

Supplementary Figure 2 – Clustal W alignments for HMW1A and HMW2A promoter regions from ON, OFF and *kan* strain sets from strains 723 (*modA2*), C486 (*modA4*) and 477 (*modA5*). Results of sequencing over HMW promoter regions encompassing the heptanucleotide repeat tracts, and aligned using MacVector version 11.0.2.

Supplementary Figure 3

a) Differential expression in 723 *modA2*

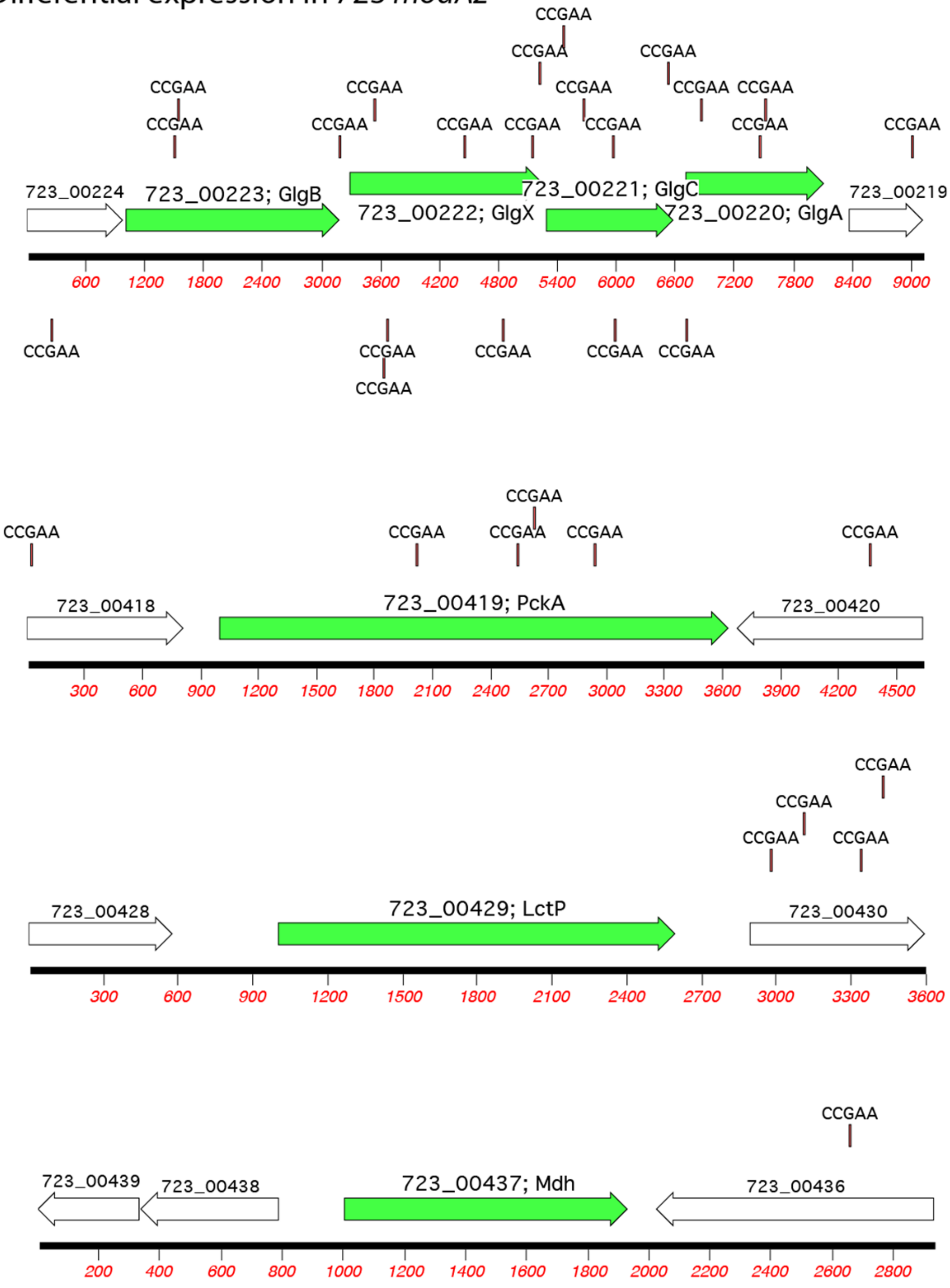

a) Differential expression in 723 *modA2* (continued)

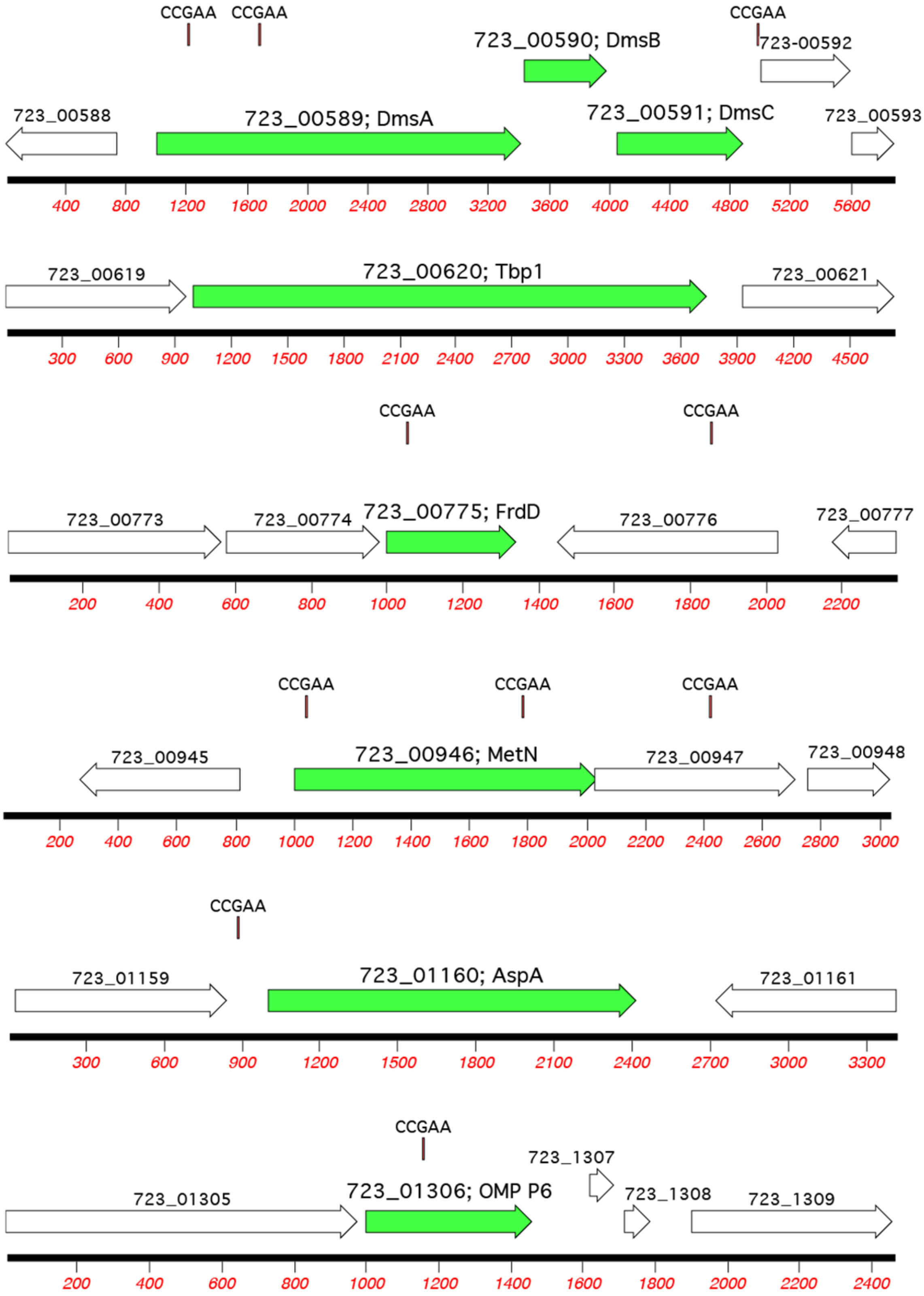

a) Differential expression in 723 *modA2* (continued)

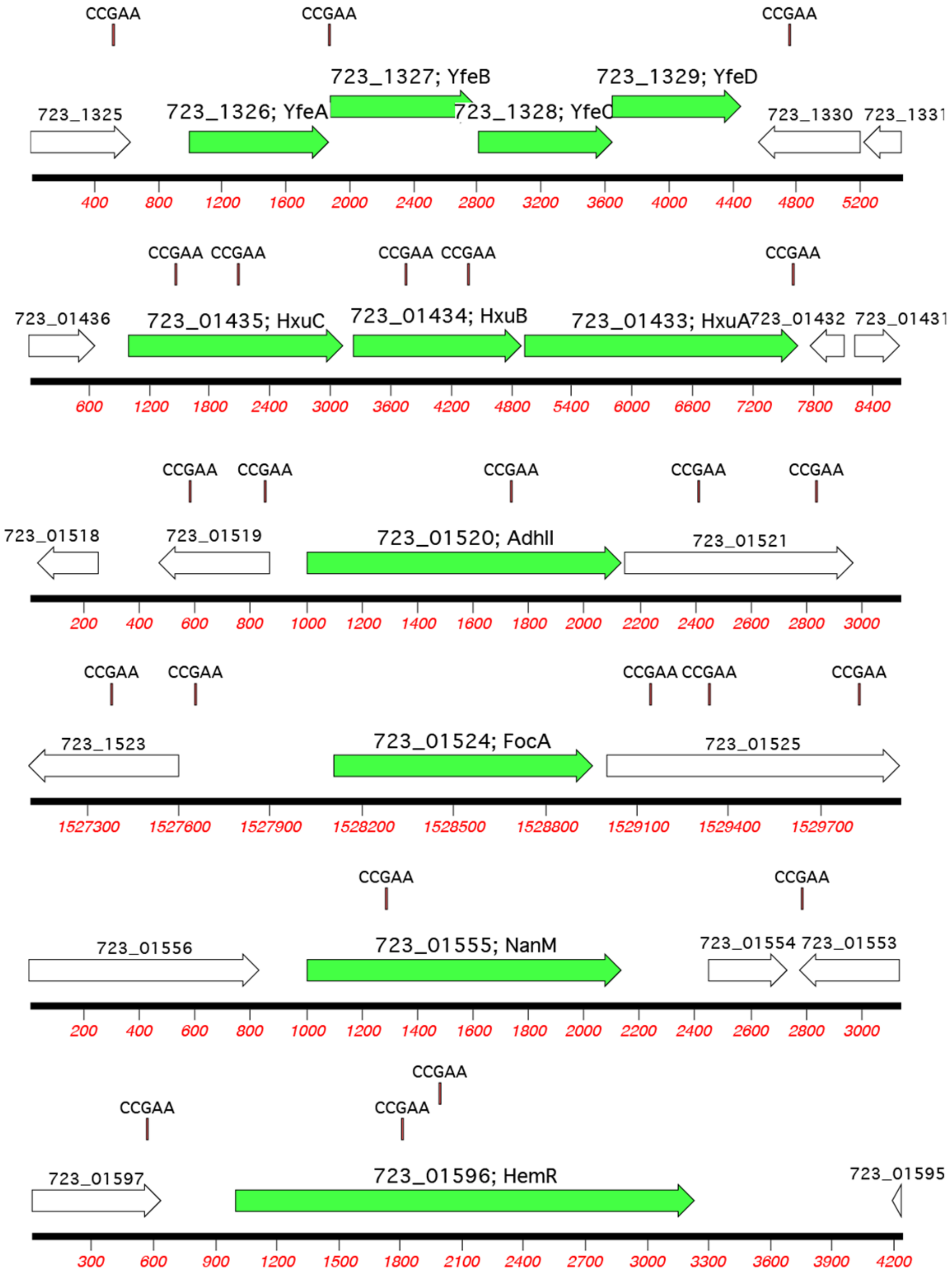

a) Differential expression in 723 *modA2* (continued)

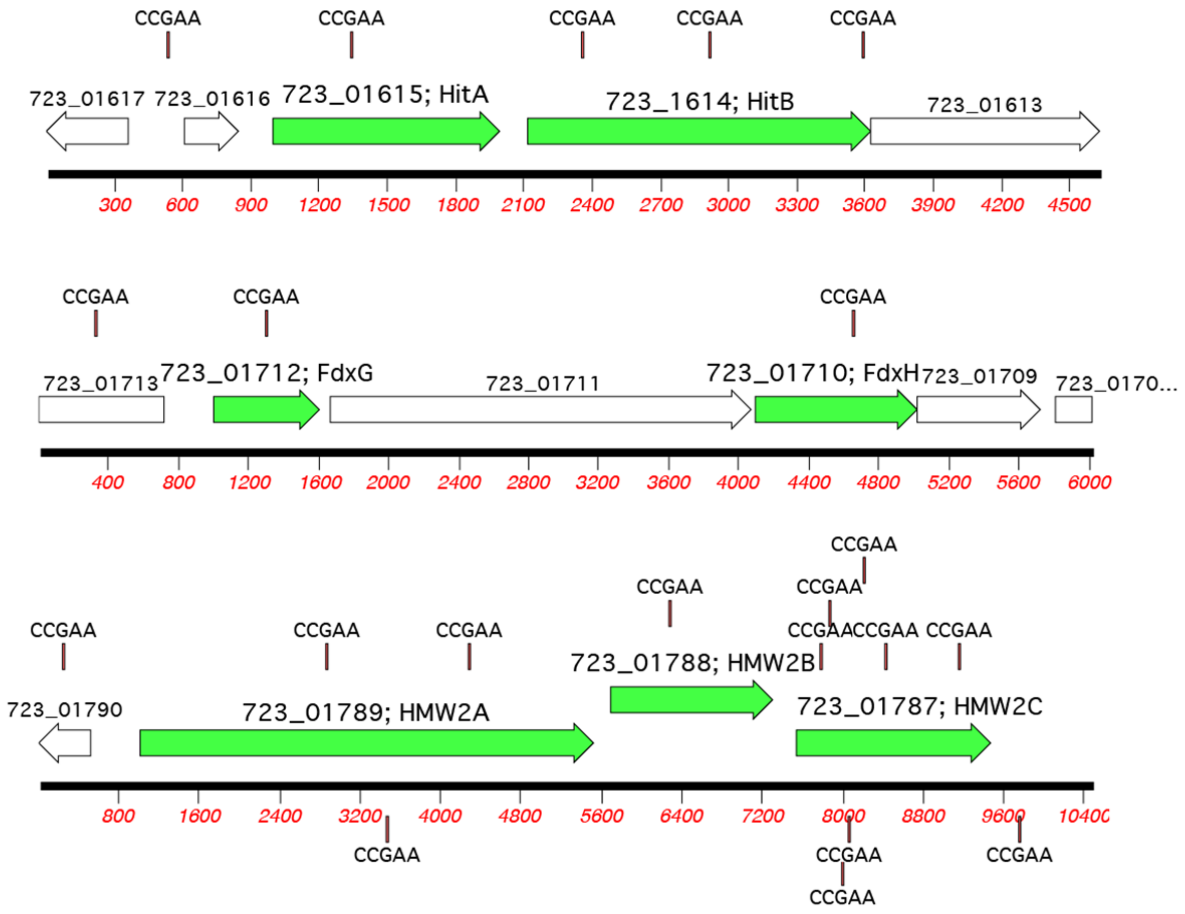

b) Differential expression in C486 *modA4*

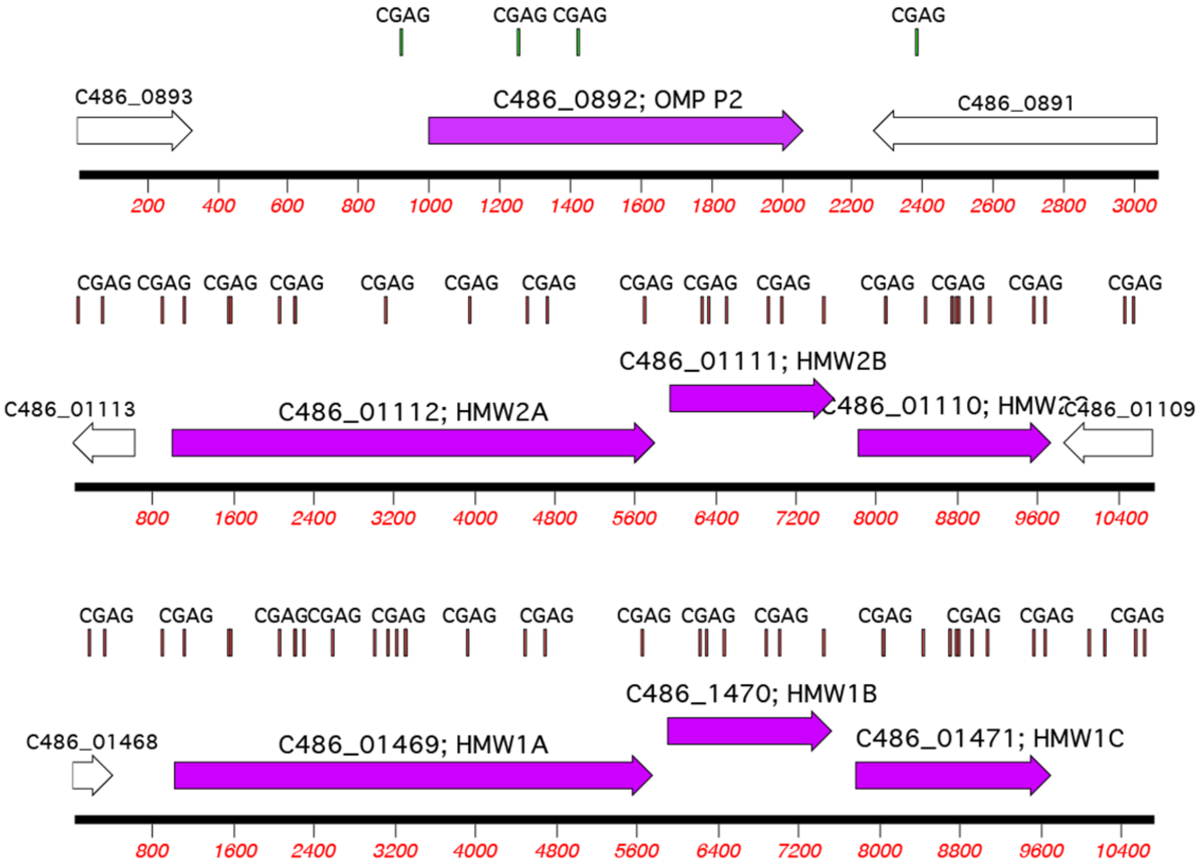

c) Differential expression in 477 *modA5*

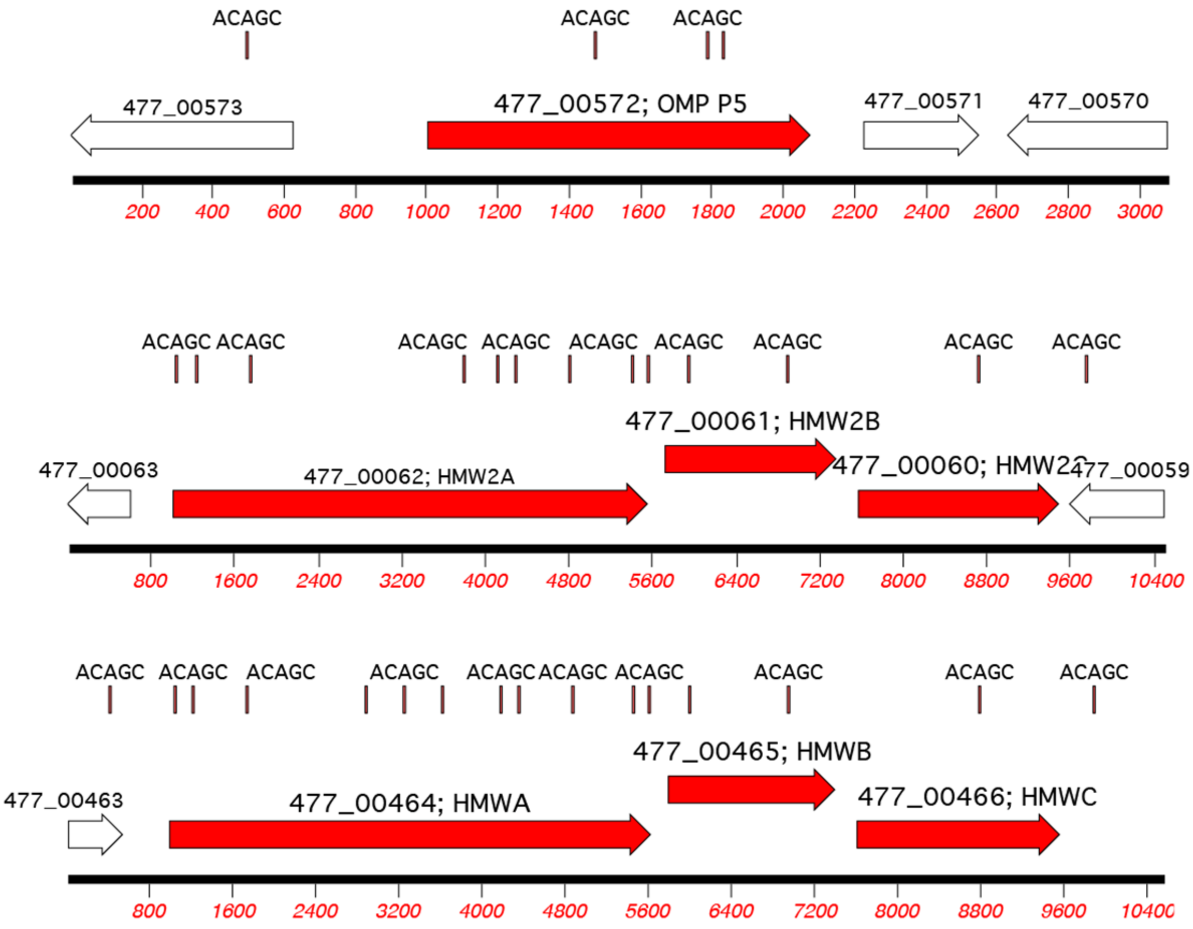

d) Differential expression in R2866 *modA10*

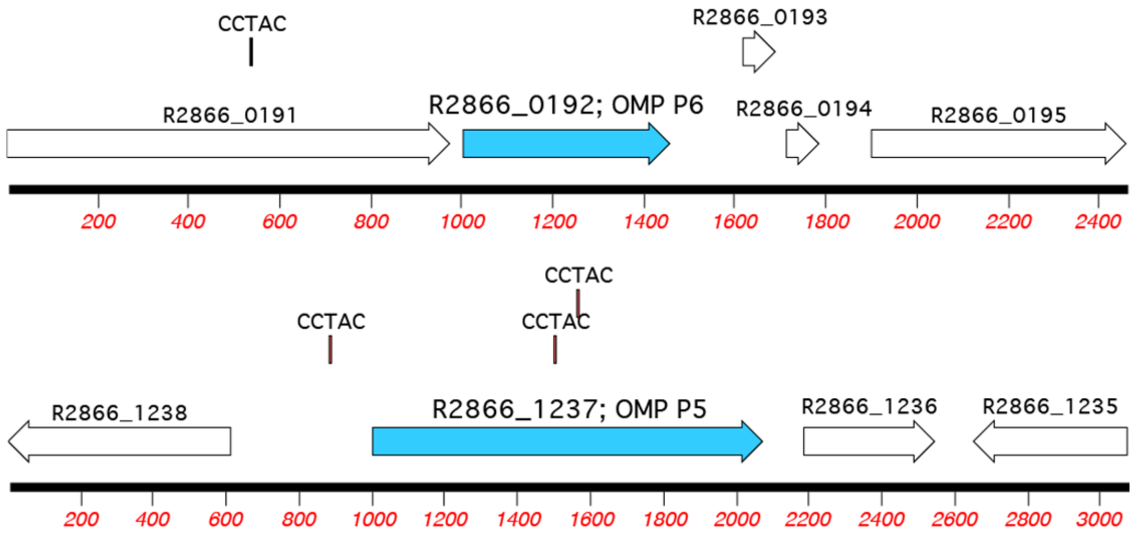

Supplementary Figure 3 – position of ModA recognition sites in relation to genes/proteins shown to be differentially regulated in *modA* ON / OFF pairs. Genes/proteins were noted as differentially expressed by iTRAQ and/or microarray. Differentially expressed gene sequences and 1000bp upstream were taken from our annotated SMRT derived genomes/NCBI GenBank. ModA recognition sequences are noted by bars above and below the sequence (ModA2 = CCGAA; ModA4 = CGAG; ModA5 = ACAGC; and ModA10 = CCTAC). Genes of interest are represented as coloured arrows, with the locus tag and gene designation given above. Flanking elements are represented as white arrows. All genes and ModA recognition sequence locations from **a)** NTHi strain 723 (*modA2*); **b)** NTHi strain C486 (*modA4*); **c)** NTHi strain 477 (*modA5*) and **d)** NTHi strain R2866 (*modA10*).

Supplementary Figure 4

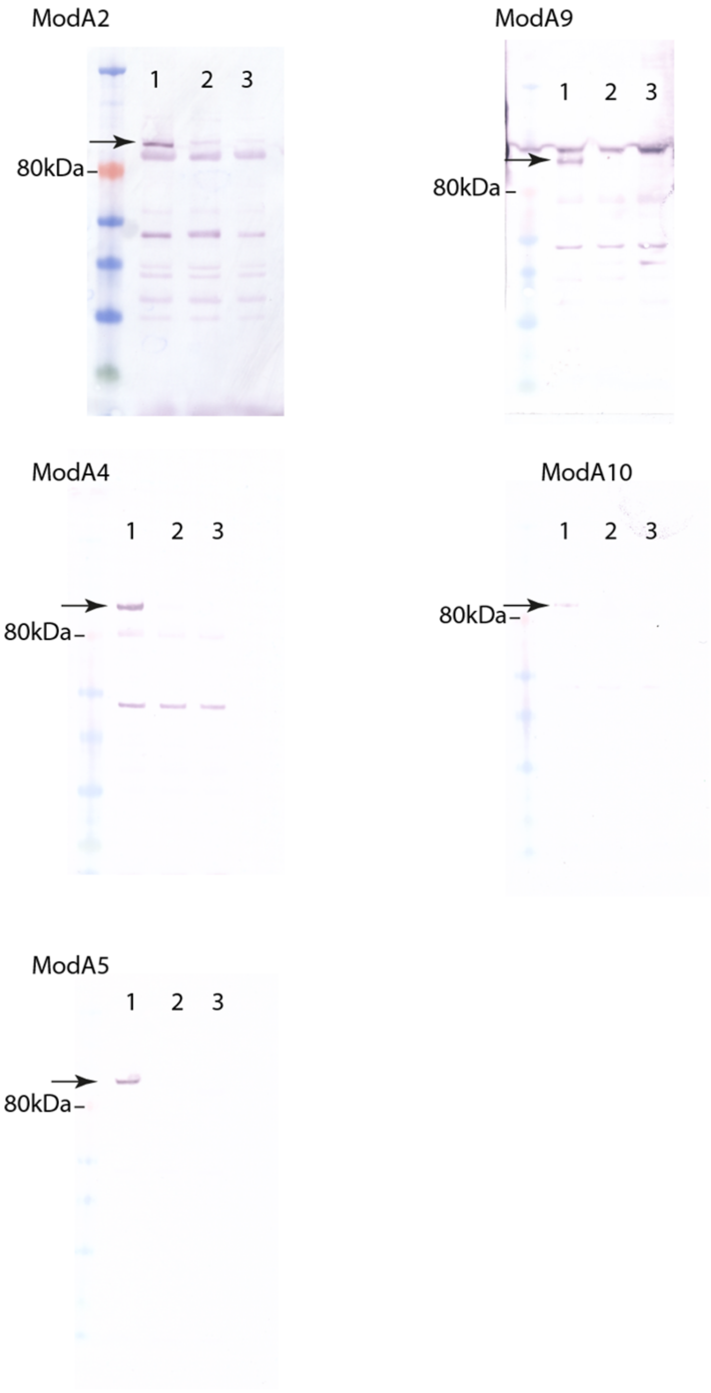

Supplementary Figure 4 – full anti-ModA western blots for NTHi strains 723 (ModA2), C486 (ModA4), 477 (ModA5) 1209 (ModA9) and R2866 (ModA10). Each blot is presented in full. Order is as presented in Figure 2; for all blots – lane 1 = ON; lane 2 = OFF; lane 3 = kanamycin knock-out strain. The band corresponding to ModA is highlighted with an arrow

Supplementary Figure 5

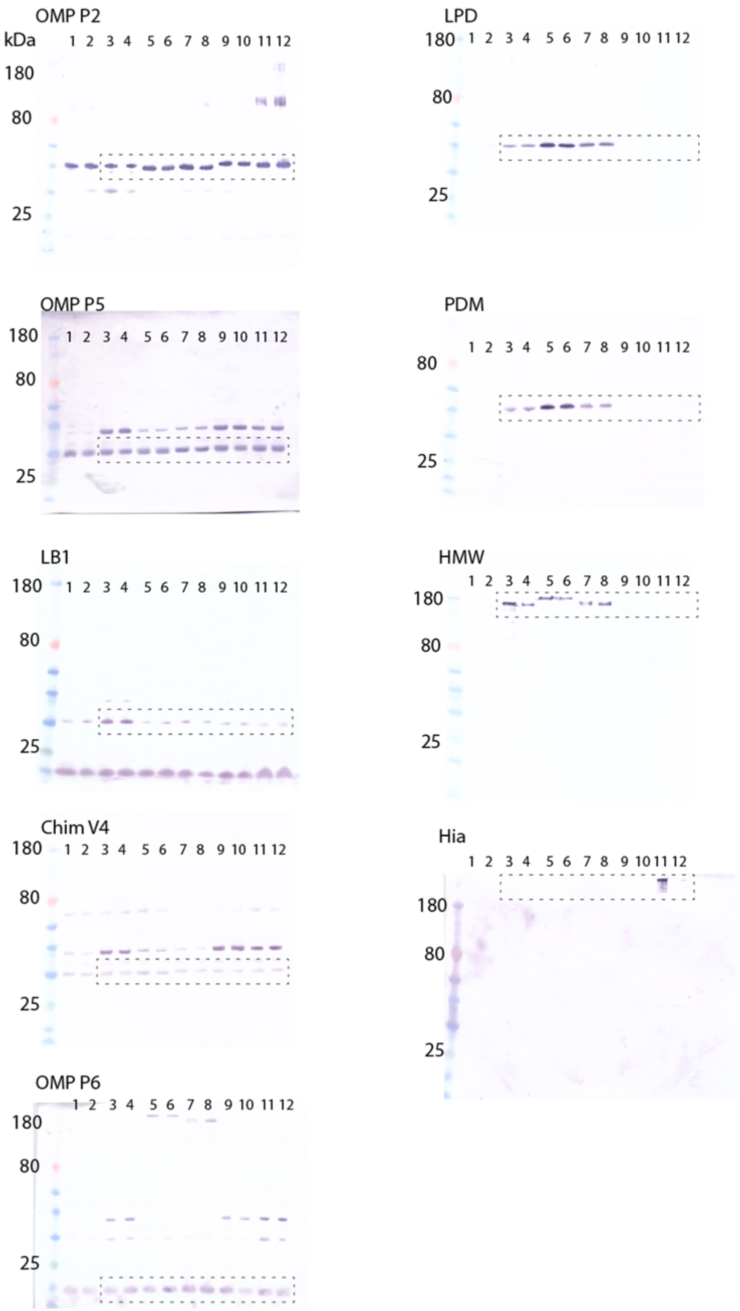

Supplementary Figure 5 – full western blots for all vaccine candidates using outer-membrane protein preparations in Figure 3. Blots are shown in full, with the representative section containing the protein of interest presented in Figure 3 boxed for clarity. Molecular weight of protein marker bands is shown to the left of each blot. The first two lanes (1&2) in all blots contain samples from *H. influenzae* strain Rd KW20, that is not considered in this work; lanes 3&4 = *modA2* ON&OFF; lanes 5&6 = *modA4* ON&OFF; lanes 6&7 = *modA5* ON&OFF; lanes 8&9 = *modA9* ON&OFF; lanes 11&12 = *modA10* ON&OFF

Supplementary Table 1 – strain collection data for NTHi *modA* allele distribution presented in Fig. 1c & 1d. NS = nasopharyngeal swab; MEE = middle ear exudate; OM = otitis media

**Data for Figure 1c**				
**NTHi isolate**	**Disease state**	**Source fluid**	***mod* allele**	**source**
001	OM prone	NS	A4	Novotny et al, 2009 [2]
002	OM prone	NS	A7	Novotny et al, 2009 [2]
006	OM prone	NS	A5	Novotny et al, 2009 [2]
013	OM prone	NS	A3	Novotny et al, 2009 [2]
017	OM prone	NS	A4	Novotny et al, 2009 [2]
043	OM prone	NS	A8	Novotny et al, 2009 [2]
047	OM prone	NS	A14	Novotny et al, 2009 [2]
050	OM prone	NS	A3	Novotny et al, 2009 [2]
052	OM prone	NS	A7	Novotny et al, 2009 [2]
059	OM-prone	NS	A7	Novotny et al, 2009 [2]
062	OM-prone	NS	A9	Novotny et al, 2009 [2]
067	OM-prone	NS	A5	Novotny et al, 2009 [2]
069	OM prone	NS	A8	Novotny et al, 2009 [2]
078	OM prone	NS	A7	Novotny et al, 2009 [2]
081	OM prone	NS	A8	Novotny et al, 2009 [2]
089	OM prone	NS	A10	Novotny et al, 2009 [2]
093	OM prone	NS	A8	Novotny et al, 2009 [2]
094	OM prone	NS	A7	Novotny et al, 2009 [2]
138	OM prone	NS	A8	Novotny et al, 2009 [2]
140	OM prone	NS	A2	Novotny et al, 2009 [2]
151	OM prone	NS	A10	Novotny et al, 2009 [2]
174	OM prone	NS	A3	Novotny et al, 2009 [2]
178	OM-prone	NS	A10	Novotny et al, 2009 [2]
182	OM-prone	NS	A5	Novotny et al, 2009 [2]
189	OM-prone	NS	A10	Novotny et al, 2009 [2]
020 L	OM prone	MEE	A10	Novotny et al, 2009 [2]
040 L	OM prone	MEE	A3	Novotny et al, 2009 [2]
075 R	OM prone	MEE	A8	Novotny et al, 2009 [2]
177 L	OM prone	MEE	A2	Novotny et al, 2009 [2]
182 L	OM prone	MEE	A5	Novotny et al, 2009 [2]
189 L	OM prone	MEE	A10	Novotny et al, 2009 [2]
187 L	OM prone	MEE	A7	Novotny et al, 2009 [2]
**Data for Figure 1d**				
**NTHi isolate**	**Disease state**	**Source fluid**	**mod allele**	**source**
314	Healthy	NS	A14	this study
341	Healthy	NS	A3	this study
353	Healthy	NS	A13	this study
354	Healthy	NS	A13	this study
355	Healthy	NS	A4	this study
359	Healthy	NS	A7	this study

389	Healthy	NS	A4	this study
390	Healthy	NS	A10	this study
395	Healthy	NS	A2	this study
396	Healthy	NS	A2	this study
397	Healthy	NS	A7	this study
398	Healthy	NS	A9	this study
402	Healthy	NS	A2	this study
412	Healthy	NS	A4	this study
414	Healthy	NS	A4	this study
452	Healthy	NS	A8	this study
484	Healthy	NS	A9	this study
490	Healthy	NS	A10	this study
491	Healthy	NS	A20	this study
497	Healthy	NS	A10	this study
503	Healthy	NS	A10	this study
515 R	Healthy	NS	A21	this study
518 R	Healthy	NS	A2	this study
525 R	Healthy	NS	A10	this study
530 L	Healthy	NS	A10	this study
538 L	Healthy	NS	A8	this study
574 R	Healthy	NS	A10	this study
577 L	Healthy	NS	A4	this study
578 L	Healthy	NS	A4	this study
591 L	Healthy	NS	A8	this study
593 R	Healthy	NS	A10	this study
597 R	Healthy	NS	A8	this study
615 L	Healthy	NS	A10	this study
624 R	Healthy	NS	A2	this study
630 R	Healthy	NS	A9	this study
639 L	Healthy	NS	A9	this study
652 L	Healthy	NS	A9	this study

Supplementary Table 2 – Minimal Inhibitory Concentration (MIC) data for a number of clinically relevant antibiotics used to treat NTHi infections

* MIC was based on previous studies with *H. influenzae* [3-6]. MICs (mg/L) are reported as the last well in which turbidity was observed following overnight growth

**Antibiotics (MIC range)***	**723 *modA2***		**C486 *modA4***		**477 *modA5***		**1209 *modA9***		**R2866 *modA10***	
	ON	OFF	ON	OFF	ON	OFF	ON	OFF	ON	OFF
Ampicillin (≤0.5->512)	0.313	0.625	0.625	0.625	0.625	0.625	0.313	0.313	>200	>200
Azithromycin (0.03–32)	0.625	0.625	0.156	0.156	0.313	0.313	0.156	0.156	0.625	0.625
Amoxillicin (0.03-16)	0.39	0.39	0.39	0.39	0.39	0.39	0.39	0.39	>250	>250
Ciprofloxacin (0.12-32)	0.008	0.008	0.008	0.008	0.008	0.008	0.004	0.004	0.008	0.008
Clindamycin (0.03-16)	3.125	3.125	0.781	0.781	0.781	0.781	1.563	1.563	1.563	1.563
Erythromycin (0.5-8)	5	5	1.25	1.25	5	2.5	2.5	2.5	5	5
Gentamicin (≤4)	2.5	2.5	1.25	1.25	1.25	1.25	5	5	5	2.5
Nalidixic Acid (2≤)	0.625	0.625	0.313	0.313	0.625	0.625	0.313	0.313	0.625	0.625

Supplementary Table 3 – genes showing > 1.4 fold up or down regulation and that are statistically significant (B-Stat >0) microarray data for the *modA2*ON / *kan* pair. [§] Gene expression was confirmed by quantitative RT-PCR in NTHi strain 723 *modA2*ON and 723 *modA2*OFF strain variants. # identified by both microarray and iTRAQ.

Gene	Microarray ID	Description	Ratio	QRT-PCR	B-Stat
**Reduced expression in the *H. influenzae* strain 723 *modA2* mutant**					
723_00381	Hflu203000743	hypothetical	-1.46		1.15
723_00559	HI1080	Amino-acid ABC transporter binding protein *tcyA*	-1.47		3.22
723_01712	NTHI0007	Formate dehydrogenase major subunit *fdxG*	-1.50		2.77
723_01520	Hflu203001461	Alcohol dehydrogenase, class III	-1.53		2.42
723_01710	NTHI0010	Formate dehydrogenase, iron-sulfur subunit *fdxH*	-1.53		1.66
723_00560	HI1079	Amino acid ABC transporter permease *tcyB*	-1.62		2.92
723_00947	HI0620.1	Methionine transport permease *metI*	-1.78		4.24
723_00429	Hflu103001790	L-lactate permease *lctP*	-1.79		3.08
723_00946	Hflu203000185	DL-methionine transporter ATP-binding subunit *metN*	-1.96	-3.56±0.741[§]	4.80
**Increased expression in the *H. influenzae* strain 723 *modA2* mutant**					
723_01433	ORF02024	Heme/hemopexin-binding precursor *hxuA*	1.45		0.86
723_01175	Hflu203001806	Nucleoside permease *nupC*	1.47		0.59
723_00220	NTHI1806	Glycogen synthase *glgA*	1.49		1.84
723_01174	Hflu203001807	Pyruvate-formate lyase-activating enzyme	1.49		3.11
723_01557	Hflu203001418	Sialic acid transporter *siaP*	1.50		1.86
723_00403	Hflu203000922	ABC-type Co2+ transport system *cbiK*	1.51		1.44
723_00222	NTHI1808	Glycogen debranching enzyme *glgX*	1.52		1.27
723_01160	HI0534	Aspartate ammonia-lyase *aspA*	1.52		2.66
723_01189	Hflu103000707	Ribose ABC transporter *rbsC*	1.53		1.58
723_00776	ORF01899	Fumarate reductase subunit D *frdD*	1.56		0.89
723_01524	HI0181	Formate transporter *focA*	1.57		1.14
723_01327	Hflu203001649	Iron (chelated) ABC transporter *yfeB*	1.61		1.32
723_00221	NTHI1807	Glucose-1-phosphate adenylyltransferase *glgC*	1.61		3.76
723_00223	NTHI1809	Glycogen branching enzyme *glgB*	1.66		2.21
723_00419	HI0809	Phosphoenolpyruvate carboxykinase *pckA*	1.69		1.00
723_01614	ORF00565	Iron (III) ABC transporter, permease protein *hitB*	1.75		4.39
723_01621	Hflu203001380	Glycerate kinase	1.81		5.06
723_01328	Hflu203001648	Iron chelated ABC transporter permease *yfeC*	1.90		1.69
723_01329	Hflu203001647	Iron chelated ABC transporter permease *yfeD*	2.08		3.98
723_00591	Hflu203000516	Anaerobic DMSO chain C *dmsC*	2.09		1.26
723_01434	Hflu103000469	Heme/hemopexin-binding *hxuB*	2.14		1.15
723_01620	NTHI0171	Gluconate family permease	2.14		5.90
723_01326	Hflu203001650	Iron chelated ABC transporter *yfeA*	2.17	2.166±0.667[§]	5.83
723_00589	Hflu203000518	Anaerobic DMSO chain A *dmsA*	2.22	2.14±0.839[§]	2.38
723_00437	HI1210	Malate dehydrogenase *mdh*	2.28		5.50
723_00590	Hflu203000517	Anaerobic DMSO chain B *dmsB*	2.65		2.67
723_01615	Hflu203001388	Iron-utilization *hitA*	4.04	2.08±0.397[§]	4.72

Supplementary Table 4

ANIMAL	EAR	Day							
		0.0	2.0	4.0	7.0	10.0	14.0	18.0	22.0
***modA2*(22)ON - all numbers = %ON**									
**#157**	**L**	87.9			82.9	90.6			#
	**R**			84.1	81.1	100.0			#
**#160**	**L**	87.9		56.5	80.3				#
	**R**								#
**#163**	**L**	87.9		82.8	81.3	77.2	#	#	#
	**R**				85.0	75.7	#	#	#
**#167**	**L**	87.9		84.8	#				
	**R**			83.3	84.0				
**#171**	**L**	87.9		84.6	83.1	#			#
	**R**			84.1	84.0		#	#	
**#166**	**L**	84.4		49.0					
	**R**			88.9					
**#181**	**L**	84.4							
	**R**		#	76.8					
**#183**	**L**	84.4		76.6					
	**R**			#					
**#185**	**L**	84.4		82.4	80.2	#	64.2	80.6	70.0
	**R**			#	32.0	34.5	71.1	81.1	#
**#190**	**L**	84.4		85.1		84.7	80.5		
	**R**			#		87.8	85.5		
***modA2*(24)OFF - all numbers = %ON**									
**#2**	**L**	7.2		78.0	81.3	70.8	#	#	#
	**R**			#	50.3	60.9	#	#	#
**#7**	**L**	7.2	68.0	#	79.3	88.4	59.0	#	#
	**R**			#	100.0	100.0	#	#	#
**#9**	**L**	7.2		5.2	56.0	#			
	**R**			74.0	74.1	76.8			
**#16**	**L**	7.2			84.3	57.0			
	**R**			#	100.0	#			
**#20**	**L**	7.2		78.6	#	#			#
	**R**				75.9	72.7	#		#
**#159**	**L**	9.8		0.0	0.0	63.9			
	**R**				0.0	81.1			
**#161**	**L**	9.8		0.0	50.0				
	**R**				20.8				
**#162**	**L**	9.8		37.6	41.7	41.1	53.0		
	**R**				82.9	74.4	#		
**#173**	**L**	9.8		0.0	#	#	#		
	**R**			#	0.0	0.0			
**#175**	**L**	9.8			60.5	65.3	47.0	#	#
	**R**				90.9	51.1	61.0	#	#

Supplementary Table 4 – Complete fragment analysis data used to generate the heat map presented in Figure 5a. Data are presented for each animal as the % *modA2*ON in the sample, from both left (L) and right (R) middle ear samples. Data from the inoculum (D+0) and all subsequent days (D+2, 4, 7, 10, 14, 18, and 22) are presented. Day 2 (D+2) is included here even though it is not included in the analysis presented in Figure 5, as only two of the D+2 samples contained sufficient CFU to attempt to generate a PCR product, and only one of these successfully produced a PCR product (animal number 7, left middle ear). White boxes indicate there was insufficient CFU in the sample to generate a PCR product; black boxes indicate the animal died/was sacrificed; # indicates a PCR product could not be generated despite there being sufficient CFU (>1000).

Supplementary Table 5 – Oligonucleotide primers used in this study

Name	Sequence
Him1F	**FAM-**ATG GCG GAC AAA GCA CCG AAG G
Him3	CAA AAA GCC GGT CAA TTT CAT CAA A
Him6	GCA GTT TAA AGG TAA AGT TAA GC
Him11	TCG TTA AAT GGA GCA AGT TC
modA-F	GAC GAC GAC AAG ATG AAG ACA GAC ATT CAA ACC G
modA-R	GAG GAG AAG CCC GGT TAT TCG CCA TCT TTT TTC TCC G
siaB-F	GAC GAC GAC AAG ATG AAA ATA ATA ATG ACA AGA ATT GCA ATT
siaB-R	GAG GAG AAG CCC GGG AAT TCT TTT GAA ATT AAA CTT TCG G
HMW1-F	CTC TTA GTA CTA GTA CAA ACC CAC
HMW2-F	CCA CTC AAA TAA ACT GGT TAA ATA TAC
HMW3-F	GCC GAG CAA ACC GCT TAT ATT GGC
HMW-R	GCA ATA TGG CGG AAA GTG GC
16SF	ACG GAG GGT GCG AGC GTT AAT C
16SR	CTG CCT TCG CCT TCG GTA TTC CT
Hflu203000185F	CAT TGC ACG TGC CTT AGC GA
Hflu203000185R	GCA GAA TAG ATT GCG TCG TTG C
Hflu203001388F	TGA AAG GGG ACA AAG TTG CG
Hflu203001388R	CAA CCG CTT GTA ATG CCA CAC
Hflu203001650F	CGC AAC GCT GGC TTG TAA CA
Hflu203001650R	TCT GCA TTG ATT GGC CAT AAA
Hflu203000518F	TTC CAT TTG TGC GGA TGC C
Hflu203000518R	CGG TCA TTT CTG TGC CAC G

Supplementary references

1 Clark, T. A. *et al.* Characterization of DNA methyltransferase specificities using single-molecule, real-time DNA sequencing. *Nucleic Acids Res.* **40**, e29 (2012).

2 Novotny, L. A. *et al.* Epitope mapping immunodominant regions of the PilA protein of nontypeable *Haemophilus influenzae* (NTHI) to facilitate the design of two novel chimeric vaccine candidates. *Vaccine* **28**, 279-289 (2009).

3 Bae, S. *et al.* Antimicrobial resistance in Haemophilus influenzae respiratory tract isolates in Korea: results of a nationwide acute respiratory infections surveillance. *Antimicrob Agents Chemother* **54**, 65-71 (2010).

4 Goldstein, F. W., Emirian, M. F., Coutrot, A. & Acar, J. F. Bacteriostatic and bactericidal activity of azithromycin against Haemophilus influenzae. *J Antimicrob Chemother* **25 Suppl A**, 25-28 (1990).

5 Harrison, C. J., Woods, C., Stout, G., Martin, B. & Selvarangan, R. Susceptibilities of Haemophilus influenzae, Streptococcus pneumoniae, including serotype 19A, and Moraxella catarrhalis paediatric isolates from 2005 to 2007 to commonly used antibiotics. *J Antimicrob Chemother* **63**, 511-519 (2009).

6 Perez-Vazquez, M., Roman, F., Aracil, B., Canton, R. & Campos, J. Laboratory detection of Haemophilus influenzae with decreased susceptibility to nalidixic acid, ciprofloxacin, levofloxacin, and moxifloxacin due to GyrA and ParC mutations. *J Clin Microbiol* **42**, 1185-1191 (2004).
